# Lung Basal Stem Cells Rapidly Repair DNA Damage Using the Error-Prone Nonhomologous End-Joining Pathway

**DOI:** 10.1371/journal.pbio.2000731

**Published:** 2017-01-26

**Authors:** Clare E. Weeden, Yunshun Chen, Stephen B. Ma, Yifang Hu, Georg Ramm, Kate D. Sutherland, Gordon K. Smyth, Marie-Liesse Asselin-Labat

**Affiliations:** 1 ACRF Stem Cells and Cancer Division, The Walter and Eliza Hall Institute of Medical Research, Parkville, Victoria, Australia; 2 Department of Medical Biology, The University of Melbourne, Parkville, Victoria, Australia; 3 Bioinformatics Division, The Walter and Eliza Hall Institute of Medical Research, Parkville, Victoria, Australia; 4 Department of Biochemistry and Molecular Biology, Monash University, Victoria, Australia; 5 Department of Mathematics and Statistics, The University of Melbourne, Parkville, Victoria, Australia; Harvard University, UNITED STATES

## Abstract

Lung squamous cell carcinoma (SqCC), the second most common subtype of lung cancer, is strongly associated with tobacco smoking and exhibits genomic instability. The cellular origins and molecular processes that contribute to SqCC formation are largely unexplored. Here we show that human basal stem cells (BSCs) isolated from heavy smokers proliferate extensively, whereas their alveolar progenitor cell counterparts have limited colony-forming capacity. We demonstrate that this difference arises in part because of the ability of BSCs to repair their DNA more efficiently than alveolar cells following ionizing radiation or chemical-induced DNA damage. Analysis of mice harbouring a mutation in the DNA-dependent protein kinase catalytic subunit (DNA-PKcs), a key enzyme in DNA damage repair by nonhomologous end joining (NHEJ), indicated that BSCs preferentially repair their DNA by this error-prone process. Interestingly, polyploidy, a phenomenon associated with genetically unstable cells, was only observed in the human BSC subset. Expression signature analysis indicated that BSCs are the likely cells of origin of human SqCC and that high levels of NHEJ genes in SqCC are correlated with increasing genomic instability. Hence, our results favour a model in which heavy smoking promotes proliferation of BSCs, and their predilection for error-prone NHEJ could lead to the high mutagenic burden that culminates in SqCC. Targeting DNA repair processes may therefore have a role in the prevention and therapy of SqCC.

## Author Summary

Human lungs are constantly exposed to inhaled chemicals that have the potential to damage cellular DNA. Lung stem cells must therefore have the ability to repair DNA damage to survive and achieve tissue homeostasis. Lung airways are composed of different types of cells, including basal cells, which have been proposed to be the stem cells of the lung. Here, we show that lung basal stem cells have a superior ability to resolve DNA damage compared to alveolar progenitor cells, thus allowing these cells to survive and proliferate after injury. Accordingly, basal stem cells isolated from patients with a long history of tobacco smoking had remarkable proliferative potential compared to those extracted from never smokers. However, we demonstrate that basal stem cells predominately use nonhomologous end joining to repair DNA double-strand breaks, a notoriously error-prone pathway. A subset of polyploid basal stem cells was observed in cigarette-smoking patients, pointing to the accumulation of genetic instability in these long-lived cells. Gene expression analyses revealed that lung squamous cell carcinoma, a subtype of lung cancer that almost exclusively occurs in smokers, carries a transcriptional fingerprint of basal cells, suggesting that lung basal stem cells could be the cells of origin of this subtype of lung cancer. We postulate that further unravelling of DNA repair in lung cells may lead to potential therapeutic targets in the prevention or treatment of lung diseases.

## Introduction

Human lungs are constantly exposed to inhaled environmental and chemical insults that have the potential to damage cellular DNA. Lung stem and progenitor cells must be capable of repairing their DNA to maintain healthy survival. The failure of stem cells to repair DNA damage can contribute to tissue loss through damage-induced cell death, whereas unfaithful DNA repair in stem cells may invoke carcinogenesis through the accumulation of genetic aberrations [1]. Lung squamous cell carcinoma (SqCC), the second most common histological subtype of lung cancer, exhibits strong genomic instability and occurs almost exclusively in smokers, with 96% of patients having a history of tobacco use [2–4]. The carcinogens present in cigarette smoke are likely responsible for the extraordinarily high mutational rate observed in SqCC compared to other cancers [4].

The early molecular events caused by tobacco exposure and the cell types in which these genetic aberrations occur to induce SqCC formation are not well known. Stem/progenitor cells are putative tumour-initiating cells because of their capacity for renewal and their longevity, allowing for accumulation of genetic lesions. Susceptibility of different lung epithelial progenitor cells to DNA damage has not been explored and could further inform the mechanisms involved in smoking-induced carcinogenesis. DNA damage encompasses alterations to bases, strand cross-links, single-strand breaks (SSBs), and double-strand breaks (DSBs). DSBs, which have been shown to arise after cigarette smoke exposure [5–7], are the most dangerous type of DNA lesion, as they can result in loss or gain of genetic information through insertions, deletions, or chromosomal translocations. DSB repair occurs through either homologous recombination (HR), a high-fidelity DNA repair mechanism, or nonhomologous end joining (NHEJ), an unfaithful mechanism that is implicated in genomic instability and tumour formation [1,8].

Different types of lung progenitor cells have been proposed in distinct anatomical regions of the lung [9]. Lung airways are composed of basal, secretory, ciliated, and neuroendocrine cells. Basal stem cells (BSCs), present only in the human cartilaginous airways or the mouse trachea [10], are located between the basement membrane and the luminal airway cells and have been proposed as stem cells of the lung [9,11–13]. The alveolar compartment is composed of alveolar type 1 and type 2 (AT1 and AT2) cells. AT2 cells have progenitor activity and can replenish both AT1 and AT2 cells following lung injury [14,15], although recent studies suggest that AT1 cells could also serve as progenitors in the mouse lung after pneumonectomy [16,17]. Different cell surface markers have been used to isolate human lung BSCs [12,18,19], but few markers allowing separation of other lung epithelial cell types have been identified [20,21].

Here we used flow cytometry to isolate BSCs, luminal (club, goblet, and ciliated) cells, and AT2 cells from fresh human proximal and distal lung tissue and showed that BSCs and AT2 cells behave as progenitor cells in an in vitro colony-forming assay. BSCs from heavy smokers had an increased proliferative potential compared to those of never smokers, whereas AT2 progenitor activity was diminished in patients with long smoking histories. To investigate this striking difference in lung stem/progenitor cell response to cigarette smoke exposure, we asked if the DNA repair mechanisms differed between the two cell types. DNA damage studies following ionizing radiation or exposure to a chemical agent demonstrated that human and mouse BSCs repair their DNA more efficiently than alveolar progenitor cells using the unfaithful NHEJ pathway, leading to cell survival and proliferation. In addition, polyploidy, a phenomenon occurring during oncogenesis [22], was only observed in the BSC subset, indicating that these cells may be more prone to transformation. Bioinformatics analyses revealed that lung SqCCs carry a transcriptional fingerprint of human lung BSCs, suggesting that BSCs may behave as the cells of origin of this subtype of lung cancer. In addition, high expression levels of key NHEJ genes in lung SqCCs are associated with increased genomic instability. Collectively, our data indicate that error-prone DNA repair is a hallmark of lung SqCC and suggest that targeting NHEJ may play a role in SqCC prevention and/or treatment.

## Results

### Isolation of Epithelial Progenitor Cells in the Human Lung

**Fig 1:**
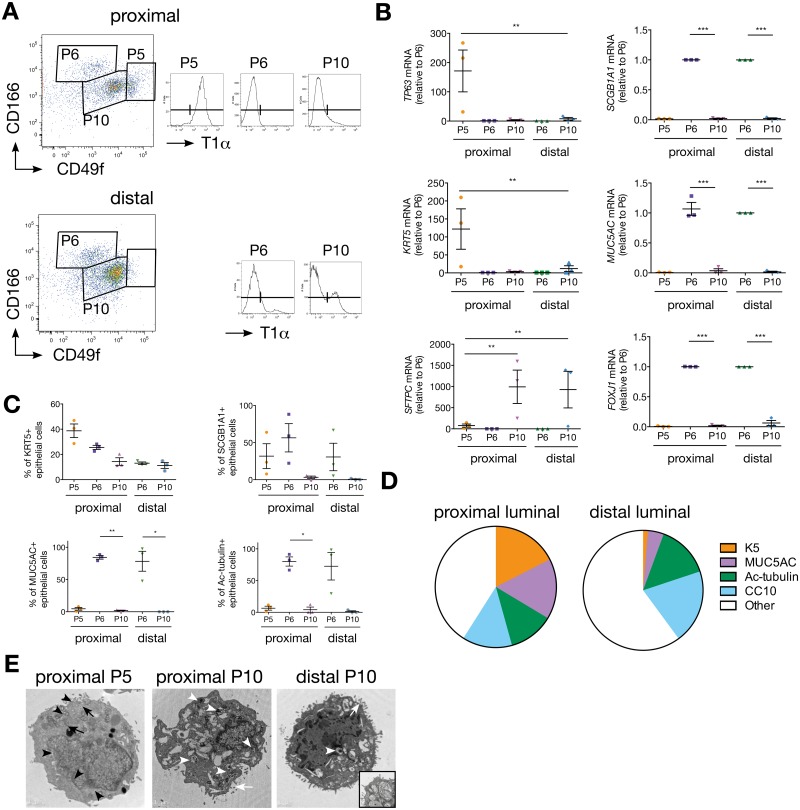
Isolation of epithelial cell subsets in the human lung. (A) EpCAM-positive, lineage- (CD45, CD31, CD140b, and CD235a) negative cells from proximal and distal lung samples are subdivided by their expression of CD166, CD49f, and T1α to collect CD49f^hi^T1α^+^CD166^mid^ (P5), CD49f^mid^T1α^-^CD166^hi^ (P6), and CD49f^mid^T1α^-^CD166^mid^ (P10) from proximal samples and P6 and P10 from distal samples. Representative image from a 63-y-old male exsmoker. *n* = 121 patients; 21–85 y old; male and female; never-, ex-, and current smokers. (B) Quantitative PCR (qPCR) analyses of lung lineage markers in sorted lung cells for *n* = 3 patients (a 65-y-old male exsmoker, a 47-y-old female exsmoker, and a 72-y-old male current smoker). Student’s *t* test. (C) Intracellular staining for differentiated lung epithelial cell markers analysed by fluorescence-activated cell sorting (FACS). *n* = 3 patients (a 47-y-old female, smoking status unknown; a 72-y-old male, smoking status unknown; and a 69-y-old male exsmoker). Student’s *t* test. (D) Pie charts showing relative cellular composition of large airway (proximal) and small airway (distal) luminal cell populations isolated from human lungs, as determined by intracellular FACS staining. *n* = 3 patients (a 47-y-old female, smoking status unknown; a 72-y-old male, smoking status unknown; and a 69-y-old male exsmoker). (E) Representative electron micrographs of proximal P5, proximal P10, and distal P10 from a 67-y-old male exsmoker. Black arrows indicate keratin filaments, and black arrowheads indicate mitochondria. White arrowheads indicate lamellar bodies, and white arrows indicate microvilli. Inset: high magnification of a lamellar body. Scale bar = 1 μm. *n* = 3 patients (a 67-y-old male exsmoker; a 61-y-old male never smoker; and a 75-y-old female, smoking status unknown). The underlying data for panels B, C, and D can be found in the S1 Data file.

Fresh human lung samples were obtained from patients undergoing lung cancer surgery and held intact in media until processing. The tissues were collected distally from the tumour sites and subdivided into proximal (containing cartilaginous airways and surrounding parenchyma) and distal (containing distal noncartilaginous airways and surrounding parenchyma) regions. A novel fluorescence-activated cell sorting (FACS) strategy was developed to deplete pre-erythrocytes, fibroblasts, and haematopoietic and endothelial cells from lung cell suspensions. Epithelial cells (EpCAM^+^, epithelial cell adhesion molecule) were then subdivided based on their expression level of CD166 (encoded by *ALCAM*), CD49f (α_6_ integrin), and T1α (also known as podoplanin). Three populations were defined in proximal samples: CD49f^hi^T1α^+^CD166^mid^ (termed P5), CD49f^mid^T1α^-^CD166^hi^ (termed P6), and CD49f^mid^T1α^-^CD166^mid^ (termed P10), and two populations in distal samples: CD49f^mid^T1α^-^CD166^hi^ (P6) and CD49f^mid^T1α^-^CD166^mid^ (termed P10) (Fig 1A and S1A Fig). These populations were consistently observed in the 121 lung samples analysed.

Quantitative PCR analysis (Fig 1B) and intracellular FACS staining (Fig 1C) of known intracellular markers of lung epithelial cells revealed that each population contained distinct cell types. P5 cells expressed high levels of the basal cell markers *TP63* and keratin 5 (*KRT5*). In contrast, both proximal and distal P6 populations contained cells that exhibited strong expression of markers of club (secretoglobin 1A1, *SCGB1A1)*, goblet (mucin 5AC, *MUC5A*C), and ciliated cells (forkhead box J1, *FOXJ1*; acetylated-tubulin) indicating this subpopulation is enriched in luminal airway cells (Fig 1B, 1C and 1D). The P10 subsets expressed high levels of the AT2 lineage marker surfactant protein C (*SFTPC*). Transmission electron microscopy further showed that the P5 population (referred to as BSC) contained cells with numerous mitochondria and keratin filaments, consistent with a basal cell phenotype [23] and their exclusive location in the proximal lung (Fig 1A and 1E). P10 populations (referred to as AT2) contained a homogenous population of AT2 cells, as evidenced by the presence of microvilli and multiple lamellar bodies (Fig 1E). These data establish that the expression of EpCAM, CD49f, CD166, and T1α is sufficient to delineate cellular compartments enriched in BSCs, luminal cells, and AT2 cells in human lung samples.

### Human Lung BSCs Are Activated by Cigarette Smoke Exposure

**Fig 2:**
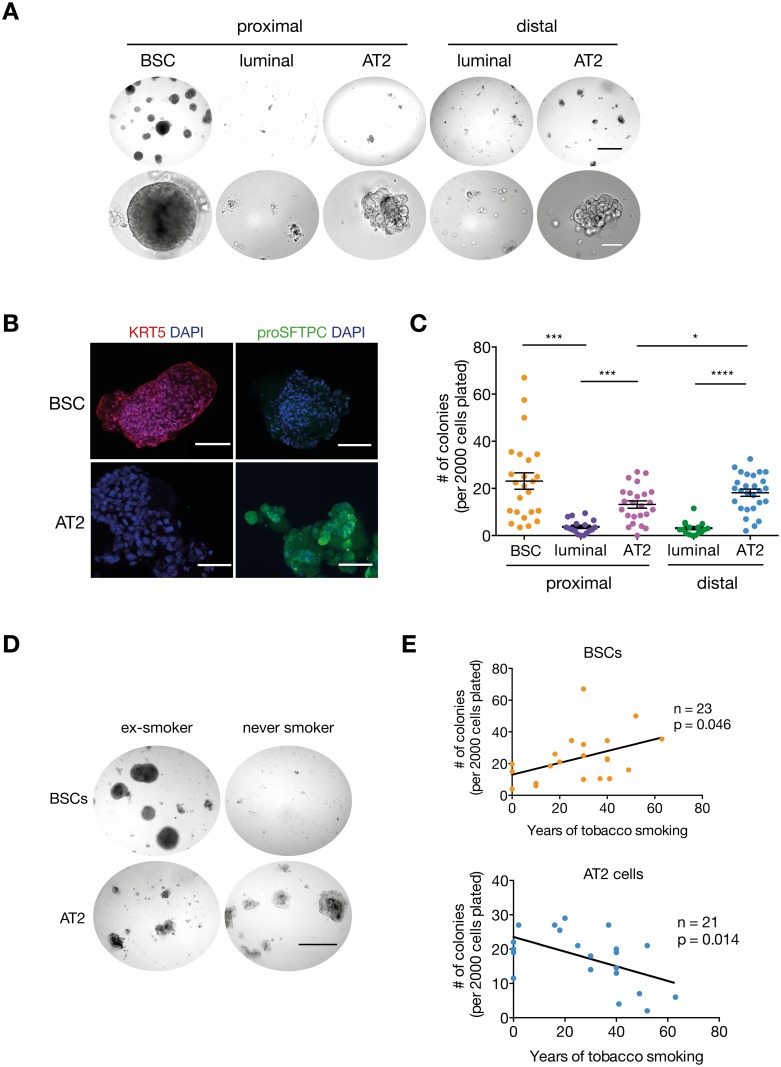
Human lung BSC and AT2 populations have progenitor activity. (A) Representative images of colonies grown from lung epithelial subsets in a 3-D in vitro assay; a 64-y-old male current smoker. Black scale bar = 500 μm; white scale bar = 100 μm. (B) Representative immunofluorescence staining of keratin 5 (KRT5) (a 57-y-old male exsmoker) and pro-surfactant protein C (proSFTPC) (a 54-y-old female exsmoker) in BSC and AT2 colonies. *n* = 13 patients; 39–78 y old; current, ex-, and never smokers. Scale bar = 100 μm. (C) Colony-forming capacity of sorted lung epithelial cells; *n* = 24 patients for proximal lung samples and *n* = 27 patients for distal lung samples; 21–85 y old; male and female; never, ex-, and current smokers. Student’s *t* test. (D) Representative images of human BSC and AT2 cell colonies from an exsmoker patient (a 57-y-old male) compared to a never-smoker patient (a 71-y-old female). Scale bar = 500 μm. (E) Linear regression analysis of the number of human BSCs (r^2^ = 0.2) or AT2 colonies (r^2^ = 0.3) versus number of years of patient tobacco smoking. *n* = 21 patients for basal colonies and *n* = 23 patients for AT2 colonies, 21–83 y old, male and female. The underlying data for panels C and E can be found in the S1 Data file.

We then assessed the colony-forming capacity of the five cellular subsets described above in a three-dimensional assay. Only BSCs and AT2 cells generated colonies that were phenotypically distinct (Fig 2A). BSCs formed clonal, hollow, spherical colonies, whilst AT2 colonies were saccular and less uniform (Fig 2A, S1B and S1C Fig). Immunostaining showed that BSC colonies maintain expression of KRT5, whilst AT2 colonies expressed SFTPC, indicating that the cells retain their lineage commitment in this culture system (Fig 2B). Distal AT2 cells had a significantly higher number of colony-forming units (CFUs) than proximal AT2 cells (Fig 2C), suggesting heterogeneity in the AT2 population between proximal and distal lung. We focused on the distal AT2 compartment (named AT2 from now on) because of its increased progenitor activity. Diversity in the basal cell compartment has also been suggested from studies in the mouse trachea, with BSCs and basal progenitor cells possessing different colony-forming capacities [24]. Heterogeneity in the BSC population may also exist in the human lung and could explain the variability in BSC CFUs observed in our study (Fig 2C). To further interrogate the diversity in the colony-forming capacity of BSCs, we investigated the association between patient tobacco-smoking history and the proliferative potential of human lung progenitor cells. Strikingly, BSCs isolated from an exsmoker patient formed numerous large colonies compared to BSCs isolated from a never smoker, which only formed a limited number of small colonies (Fig 2D). Conversely, AT2 cells isolated from an exsmoker had reduced colony-forming capacity compared to AT2 cells from a never smoker that formed multiple large saccular colonies (Fig 2D). Linear correlation analysis demonstrated a positive correlation between years of smoking and the number of BSC CFUs, whereas tobacco exposure was inversely correlated with the number of AT2 cell CFUs (Fig 2E). The number of years since a patient had quit smoking, patient age, and patient sex did not correlate with colony-forming capacity (S2A, S2B and S2C Fig). These data demonstrate that exposure to cigarette smoke activates lung BSCs yet impairs AT2 cells and that this effect is maintained after smoking cessation.

### Human and Mouse BSCs Possess Increased DNA Repair Capacity

**Fig 3:**
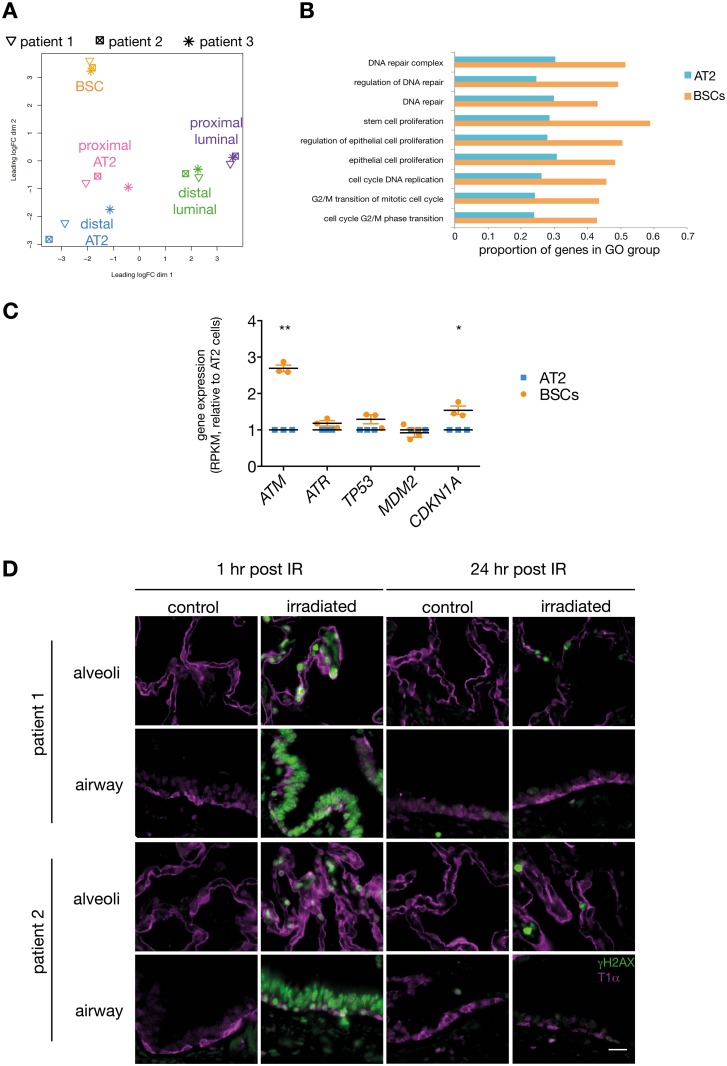
Human lung BSCs sustain less DNA damage than alveolar progenitor cells. (A) Multidimensional scaling plot of expression profiles of human lung epithelial subsets from three patients (a 64-y-old male exsmoker, an 83-y-old male exsmoker, and a 53-y-old male current smoker). Distances represent the leading log2-fold change. (B) Gene ontology (GO) terms associated with DNA repair or the cell cycle are significantly up-regulated in BSCs compared to AT2 cells by rotation gene set tests (ROAST) (*p* < 0.02). Each pair of bars corresponds to a relevant (GO) term. Bars show the proportion of genes associated with the GO term that are more highly expressed in BSCs (orange) or in AT2 cells (blue), as determined by limma’s roast function. (C) Expression of key genes in the DNA repair pathway in BSCs relative to AT2 cells. RPKM, reads per kilobase per million mapped reads. *n* = 3 patients; a 64-y-old male exsmoker, an 83-y-old male exsmoker, and a 53-y-old male current smoker. Paired *t* test. (D) Immunofluorescence staining of γH2AX (green) and T1α (purple) in whole human lung fragments that are nonirradiated (control) or 1 or 24 h post irradiation (6 Gy). *n* = 2 patients (a 60-y-old female never smoker and a 78-y-old female never smoker). Scale bar = 20 μm. The underlying data for panels A, B, and C can be found in the S1 Data file.

To investigate the molecular mechanisms driving the differential response of AT2 cells and BSCs to cigarette smoking, we performed RNA sequencing on freshly isolated cells from current and exsmokers. Unsupervised clustering showed that each population was molecularly distinct (Fig 3A and S3A Fig). Gene ontology analyses revealed that cell cycle and DNA repair genes were up-regulated in BSCs compared to AT2 cells (Fig 3B). Human BSCs also expressed high levels of telomere maintenance genes, including *TERT* (S3B Fig), and were found to have longer telomeres than AT2 cells (S3C Fig). Given that both active DNA repair and telomere maintenance are properties of stem cells [25,26], these findings align with mouse studies and confirm that BSCs have greater stem cell-like characteristics than the AT2 progenitor cells [11,27]. BSCs exhibited up-regulation of key genes that control the activation of DNA repair pathways, including *ATM* (ataxia telangiectasia mutated) [28] (Fig 3C), suggesting that BSCs may have an enhanced ability to respond to DNA damage. To evaluate the sensitivity of human lung stem and progenitor cells to DNA damage, we subjected fresh human lung tissue to ionizing radiation (IR) and assessed the presence of DSBs over time by immunofluorescence analysis of phosphorylated histone 2AX (γH2AX), an early marker of DSBs. Strong γH2AX staining was observed in both the alveolar and BSC compartment 1 h after IR exposure (Fig 3D). However, DSBs were resolved in the basal cell compartment 24 h post IR, whereas γH2AX staining was still detected in the alveolar region at this time point (Fig 3D). These results suggest that human BSCs exhibit an increased capacity to repair their DNA following IR compared to alveolar cells.

**Fig 4:**
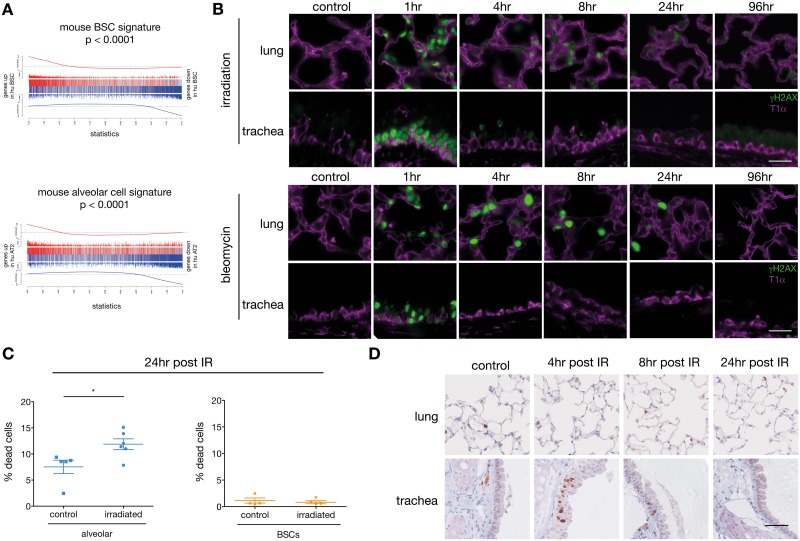
Mouse tracheal BSCs are less sensitive than alveolar cells to DNA damage-induced cell death. (A) Barcode plots showing strong correlation of mouse and human expression signatures. The upper panel correlates the mouse tracheal BSC signature with that of human lung BSCs; the lower panel correlates the mouse alveolar cell signature with that of human AT2 cells. For each plot, genes are ordered left to right from most up-regulated to most down-regulated in human BSCs or AT2 cells, relative to all other cell populations. Positive mouse signature genes are marked with red vertical bars, and negative mouse signature genes with blue. Variable-height vertical bars show log-fold changes for the mouse signature genes. Worms show relative enrichment of mouse genes in the human ranked list. Rotation gene set tests give *p* = 0.0001 for each plot. (B) Immunofluorescence staining of γH2AX and T1α in alveoli or trachea of wild-type (WT) mice that are either nonirradiated or 1, 4, 8, 24, or 96 h post irradiation (6 Gy, top panel) or saline injected or 1, 4, 8, 24 or 96 h post bleomycin injection (40 mg/kg intravenously, bottom panel). T1α marks alveolar type 1 cells in the lung and BSCs in the trachea. Representative images of *n* = 3 mice at each time point. Scale bar = 20 μm. (C) FACS detection of dead cells in lung alveolar (EpCAM^+^) or tracheal BSCs (T1α^+^) isolated from WT mice 24 h post irradiation (6 Gy). *n* = 4–6 mice per group. Student’s *t* test. (D) Immunohistochemistry staining of Ki67 in alveoli or trachea of WT mice control or 4 h, 8 h, or 24 h post irradiation (6 Gy). Representative images for one of *n* = 3 mice at each time point. Scale bar = 20 μm. The underlying data for panel C can be found in the S1 Data file.

Given that the transcriptomic analysis and the study of the response to IR were performed on human samples from patients with different smoking histories, we sought to determine whether this striking difference in gene expression profile and DNA damage response of human BSCs and AT2 cells was acquired as a result of chronic cigarette smoke exposure or if it was an inherent property of the cells. Healthy lungs from never smokers are difficult to obtain; hence, we performed RNA-seq transcriptional profiling on mouse tracheal BSCs and lung alveolar cells (S4A Fig). Gene expression profiles of mouse BSCs and alveolar cells significantly correlated with their human counterparts, indicating that the transcriptome of lung stem/progenitor cells is highly conserved between species (Fig 4A). Consistent with the human data, we observed that DNA repair genes and cell cycle genes were up-regulated in mouse BSCs compared to alveolar cells (S4B Fig), suggesting that both mouse and human lung BSCs may be intrinsically positioned to repair DNA damage. γH2AX immunofluorescence staining of mice subjected to IR revealed that both BSCs and alveolar cells had DSBs 1 h post IR, and this was detected in a dose-dependent manner (Fig 4B, S4C Fig). However, γH2AX expression was resolved in BSCs 4 h post IR, whilst it was still strongly detected in the alveolar compartment 24 h after IR (Fig 4B). Similar results were observed when the mice were injected with bleomycin, a DNA-damaging agent known to induce DSBs [29] (Fig 4B). The sensitivity of mouse alveolar cells to IR was reflected in increased apoptosis that was not observed in BSCs (Fig 4C, S4D and S4E Fig). Whilst it is possible that BSCs undergo cell senescence, BSCs proliferated 4 h and 8 h after IR (Fig 4D), suggesting that senescence in these cells is unlikely. Therefore, lung BSCs have superior DNA repair capabilities leading to cell survival and proliferation that is conserved across species, while alveolar cells exhibit limited DNA repair capacity resulting in DNA damage-induced cell death.

### BSCs Use NHEJ to Repair DNA DSBs

**Fig 5:**
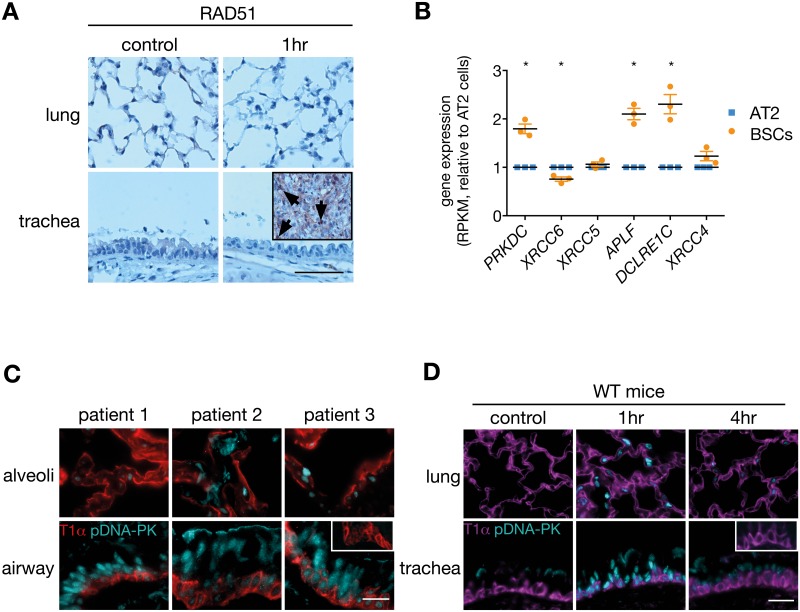
Human and mouse BSCs express markers of nonhomologous end joining. (A) Immunohistochemistry for RAD51, an early marker of homologous recombination, on WT mouse trachea and lung 1 h post γ-irradiation (6 Gy). The insert is a positive control, a mammary tumour from a MMTV-cre;Brca1^fl/fl^p53^+/-^ mouse. Black arrows indicate RAD51-positive nuclei. Representative images from *n* = 3 mice at each time point. Scale bar = 100 μm. (B) Expression of key genes in the NHEJ repair pathway in human BSCs and AT2 cells. *n* = 3 patients (a 64-y-old male exsmoker, an 83-y-old male exsmoker, and a 53-y-old male current smoker). RPKM, reads per kilobase per million mapped reads. Paired *t* test. (C) Immunofluorescence staining of phospho-DNA-PKcs and T1α in human airways and alveoli of three patients. Patient 1, a 56-y-old male smoker; patient 2, a 69-y-old female exsmoker; patient 3, a 70-y-old male smoker. Inset, isotype control. Scale bar = 20 μm. (D) Immunofluorescence staining of phospho-DNA-PKcs and T1α in trachea and lung of WT mice following IR (6 Gy). Representative images of one of *n* = 3 mice at each time point. Inset, isotype control. Scale bar = 20 μm. The underlying data for panel B can be found in the S1 Data file.

Our results show that BSCs have a prompt and enhanced ability to repair DSBs compared to alveolar cells in vivo. NHEJ is a rapid DSB repair process that occurs in all phases of the cell cycle, while HR functions only in actively cycling cells [30]. Given that the majority of BSCs and alveolar cells reside in the G0/G1 phase of the cell cycle (S4D Fig) and that RAD51, an early marker of HR, was not detected in mouse BSCs after IR (Fig 5A), we interrogated whether BSCs preferentially used NHEJ to repair DSBs. Genes regulating NHEJ were found to be up-regulated in human and mouse BSCs compared to alveolar cells (Fig 5B and S5A Fig), including *PRKDC* that encodes for the DNA-dependent protein kinase catalytic subunit (DNA-PKcs), a necessary enzyme for the initiation of NHEJ [31]. Activation of DNA-PKcs was observed in lung samples from cigarette-smoking patients and in irradiated mouse lung and trachea as detected by immunofluorescence staining of phosphorylated DNA-PKcs (Fig 5C and 5D).

**Fig 6:**
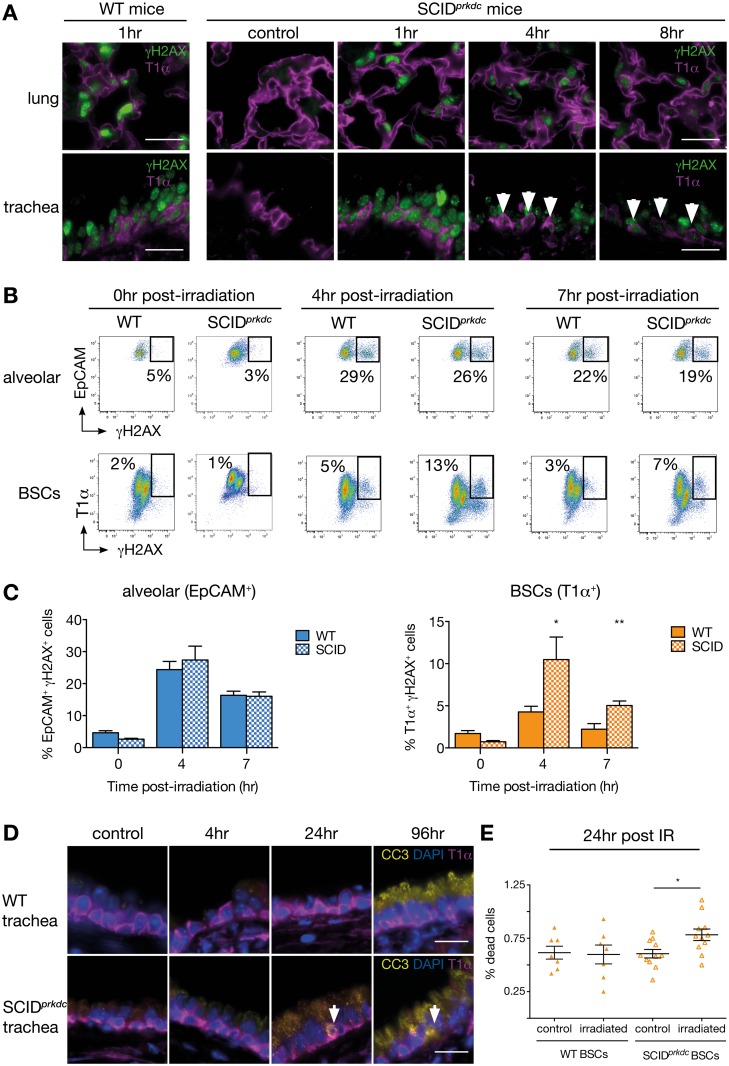
BSCs use error-prone nonhomologous end joining to repair DNA double-strand breaks. (A) Immunofluorescence staining of γH2AX and T1α in the lungs and tracheas of WT and SCID^*prkdc*^ mice that are nonirradiated or 1, 4 or 8 h post irradiation (6 Gy). Representative images of *n* = 3 mice at each time point. Arrows indicate γH2AX^+^ T1α^+^ BSCs. Scale bar = 20 μm. (B) Representative FACS plots showing the expression of γH2AX in EpCAM^+^ lung epithelial cells and T1α^+^ tracheal BSCs in WT and SCID^*prkdc*^ mice 0, 4, and 7 h following IR (6 Gy). The timing corresponds to the number of hours between time of irradiation and generation of single-cell suspension for FACS analysis. (C) Percentage of γH2AX-positive cells in WT and SCID^*prkdc*^ mice in EpCAM^+^ lung epithelial cells and T1α^+^ tracheal BSCs 0, 4, and 7 h following irradiation. *n* = 6 animals per group. Student’s *t* test. The timing corresponds to the number of hours between time of irradiation and generation of single-cell suspension for FACS analysis. (D) Immunofluorescence staining of cleaved caspase 3 (CC3), T1α, and 4′,6-diamidino-2-phenylindole (DAPI) in WT and SCID^*prkdc*^ tracheas that are nonirradiated or 4, 24, or 96 h post irradiation (6 Gy). Representative images of *n* = 3 mice at each time point. Arrows indicate CC3^+^ T1α^+^ BSCs. Scale bar = 20 μm. (E) FACS detection of cells in subG1 in tracheal BSCs (T1α^+^) cells isolated from WT or SCID^*prkdc*^ mice 24 h post irradiation (6 Gy). *n* = 7 mice for WT mice and *n* = 12 for SCID^*prkdc*^ mice. Student’s *t* test. The underlying data for panels C and E can be found in the S1 Data file.

To assess whether BSCs use NHEJ to repair their DNA, we analysed severe combined immune deficiency (SCID^*Prkdc*^) mice, that have a mutation in *Prkdc*, leading to a 50% reduction in DNA-PKcs activity and impaired NHEJ [32]. Identical levels of γH2AX were induced in the respiratory system of wild-type (WT) and SCID^*prkdc*^ mice 1 h after IR (Fig 6A). Strikingly, DSBs were still detected in tracheas of SCID^*prkdc*^ mice 8 h post IR, whereas DSBs were completely resolved in WT tracheas at this time point, indicating that BSCs use NHEJ to rapidly repair their DNA following damage (Figs 4B and 6A). Quantification by flow cytometry confirmed that BSCs from SCID^*prkdc*^ mice exhibited delayed DSB repair after IR compared to WT mice, whilst the levels of γH2AX in the alveolar compartment were not affected by reduced DNA-PKcs activity (Fig 6B and 6C). Treatment with bleomycin similarly resulted in delayed DSB repair in BSCs isolated from SCID^*prkdc*^ mice compared to WT mice, whereas knock-down of DNA-PKcs activity did not alter γH2AX expression in AT2 cells (S5B and S5C Fig). The impaired ability of BSCs from SCID^*prkdc*^ mice to repair their DNA was associated with an increase in cell death following IR that was not observed in WT BSCs (Fig 6D and 6E). In addition, alveolar cells isolated from WT and SCID^*prkdc*^ mice had similar levels of apoptosis (S5D Fig). These data establish that BSCs predominantly use the NHEJ pathway to repair DSBs to maintain cell survival and proliferation, whilst lung alveolar progenitor cells have reduced NHEJ activity.

### BSCs Are the Candidate Cells of Origin of Lung SqCC

**Fig 7:**
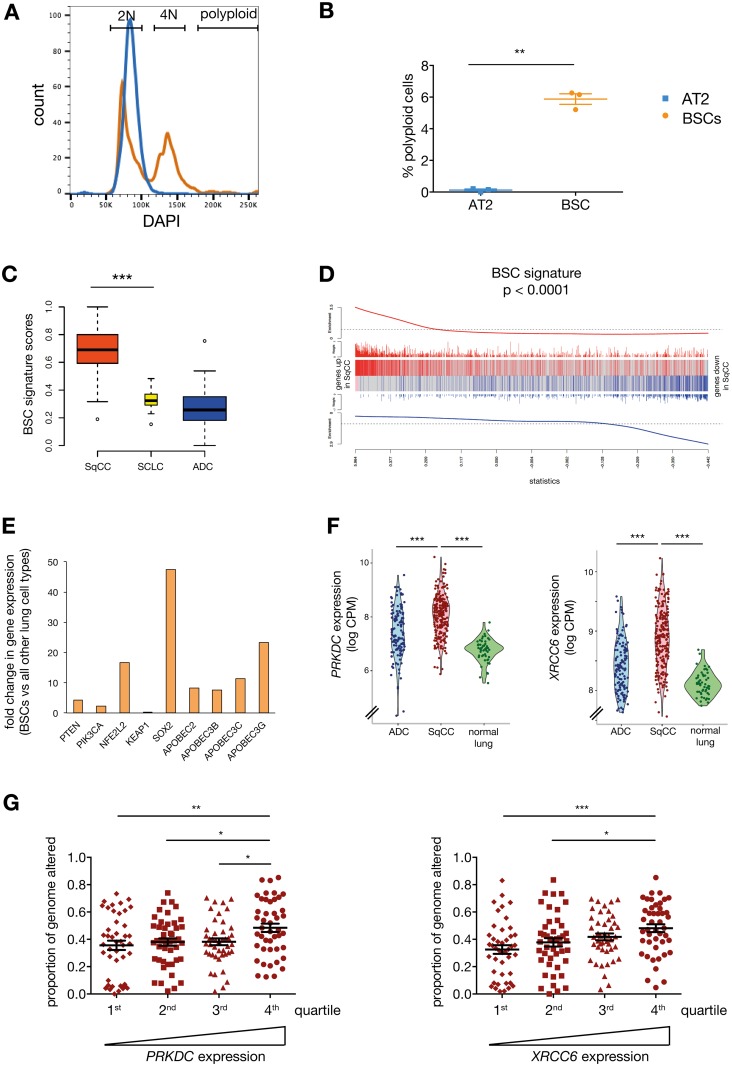
Human lung BSCs are the putative cells of origin of lung squamous cell carcinoma. (A) Representative histogram of intracellular DAPI staining of BSCs and AT2 cells isolated from a 56-y-old male exsmoker patient. Gates indicate 2N, 4N, and polyploid cells. (B) Proportion of polyploidy cells in the BSC and AT2 subsets. *n* = 3 patients (a 69-y-old female exsmoker, a 56-y-old male exsmoker, and a 70-y-old female exsmoker). Paired *t* test. (C) Boxplots of human lung BSC expression scores by lung tumour subtypes (ADC, adenocarcinoma; SCLC, small cell lung cancer; SqCC, squamous cell carcinoma). The width of each box indicates the sample size. (D) Barcode plot showing strong correlation of the human lung BSC expression signature with that of SqCCs (ROAST *p* = 0.0001). Genes are sorted left to right from most up- to most down-regulated in SqCC relative to all other cancer subtypes. Positive BSC signature genes are marked with vertical red bars, and negative signatures genes are marked in blue. Variable-height bars show log-fold-change strength for each signature gene. (E) Fold changes in the expression of genes frequently altered in lung SqCC between human BSCs and other human lung epithelial cell types. *n* = 3 patients (a 64-y-old male exsmoker, an 83-y-old male exsmoker, and a 53-y-old male current smoker). (F) Violin plots showing expression levels of *PRKDC* and *XRCC6* in normal lung tissue (*n* = 54), lung ADCs (*n* = 125), and lung SqCCs (*n* = 224) from The Cancer Genome Atlas (TCGA). Violin bodies show log2 counts per million (log CPM) expression values as smoothed densities. All pairwise *p*-values are <10^−6^ by moderated *t* tests. (G) Proportion of genome altered versus *PRKDC* and *XRCC6* expression levels in TCGA lung SqCC data (*n* = 179). Expression levels are split into quartiles. Significance was determined by Student’s *t* tests. The underlying data for panels A, B, C, D, E, and G can be found in the S1 Data file.

**Fig 8:**
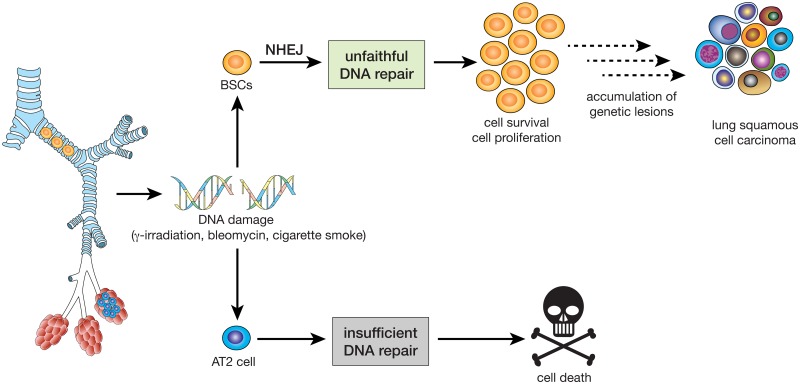
Lung BSCs have a rapid yet error-prone response to DNA damage through nonhomologous end joining (NHEJ). This allows the cells to continue to survive and proliferate after DNA injury and may eventually result in the accumulation of genetic lesions that lead to SqCC formation. In contrast, AT2 progenitor cells have an insufficient response to DNA damage, resulting in cell death.

Use of the error-prone NHEJ repair pathway has been associated with increased genetic alterations and genomic instability [1]. Interestingly, polyploid cells were detected in the human BSC subset isolated from exsmokers, whereas no such population was present in AT2 cells (Fig 7A and 7B), suggesting that a proportion of BSCs have increased genetic instability compared to AT2 cell progenitors. These results led us to investigate the involvement of BSCs in lung carcinogenesis. We used expression signature analysis to relate the transcriptome of human lung cell subsets to expression profiles of tumours available from The Clinical Lung Cancer Project (S6A Fig) [33]. We found that the expression profile of human lung BSCs was strongly associated with that of SqCC (Fig 7C and 7D). No correlation was observed between BSCs and other lung cancer subtypes, nor did any other human epithelial cell subset associate with SqCC (S6B–S6E Fig), suggesting BSCs as the putative cells of origin of SqCC. We noticed that human BSCs express higher levels of genes known to be frequently altered in SqCC, such as *NFE2L2*, *SOX2*, and *PTEN* [3,34], compared to the other lung epithelial subsets (Fig 7E). Additionally, BSCs expressed high levels of *APOBEC* cytidine deaminase genes (Fig 7E), which could explain the APOBEC signature observed in lung SqCC [4]. Interestingly, lung SqCCs express higher levels of DNA repair and cell proliferation genes compared to lung adenocarcinoma, a cancer that arises from lung AT2 cells (S7A Fig) [35,36]. Analysis of the RNA-seq data from The Cancer Genome Atlas data [3,37] showed that key NHEJ genes such as *PRKDC* and *XRCC6* are expressed at higher levels in lung SqCC compared to lung adenocarcinomas and normal lung tissue (Fig 7F and S7B Fig). Strikingly, high expression of *PRKDC* or *XRCC6* in lung SqCC was found to be associated with increased genomic instability (Fig 7G). These data suggest that DNA repair by NHEJ could prove to be a hallmark of lung SqCC. We propose that the use of NHEJ by BSCs could lead to the accumulation of genetic alterations that may culminate in SqCC formation in cigarette-smoking patients (Fig 8), although this hypothesis will need to be validated with functional studies in vivo.

## Discussion

In this study, we used a novel combination of cell surface markers to simultaneously isolate distinct human lung epithelial cell populations and observed that lung BSCs have more stem cell-like characteristics than AT2 progenitor cells. BSCs were found to have longer telomeres and a superior ability to repair DSBs compared to AT2 cells. Our study provides new evidence to indicate that adult lung stem cells have developed more efficient DNA repair mechanisms than differentiated cells to promote cell survival and tissue repair. Consistently, haematopoietic stem cells appear more resistant to IR-induced cell death than myeloid progenitors and were found to activate NHEJ to repair their DNA [38]. NHEJ has also been proposed as a mechanism for DSB repair in breast and hair follicle bulge stem cells [39,40] as a process to evade apoptosis and ensure stem cell longevity.

Cigarette smoke contains a complex mix of carcinogens and toxins that cause DNA damage, including oxidative base damage, the formation of DNA adducts, SSBs, and DSBs [7,41,42]. The observation that lung BSCs have a greater capacity to repair DNA damage compared to alveolar progenitor cells may explain the varied smoking-induced pathologies observed in specific anatomical regions of the lung. Loss of AT2 cells has been identified as a mechanism participating in the pathogenesis of idiopathic pulmonary fibrosis and emphysema-like diseases [43,44]. Different genetic mutations have been associated with the development of these diseases, including aberrations in telomere maintenance genes, *SFTPC*, *MUC5B*, and alpha-1 anti-trypsin [45–47]. Our data showing that AT2 cells are highly sensitive to DNA damage, leading to increased cell death and reduced colony-forming capacity, suggest a novel molecular mechanism that may participate in tobacco smoking-induced emphysema. They also provide further supporting evidence that epithelial cell dysfunction plays a role in the pathogenesis of degenerative lung diseases. Radiation therapy is frequently used in lung cancer patients yet is often associated with damage of surrounding normal tissue, resulting in reduced quality of life [48]. Our observation that AT2 progenitor cells have limited DSB repair capacity and increased cell death following IR may also provide insights into the adverse loss of alveolar cells and radiation-induced fibrosis following γ-irradiation.

We observed that BSCs isolated from heavy tobacco users are drastically more proliferative than those from never-smoker patients, which is consistent with the basal cell hyperplasia frequently observed in cigarette smokers [23,49]. Multiple mechanisms most likely account for the activation of BSC proliferation following exposure to tobacco smoke. Firstly, endogenous levels of reactive oxygen species (ROS) have been shown to influence the proliferative capacity of lung cells [18], and elevated ROS levels, like those induced by cigarette smoke exposure, could participate in the higher proliferative potential of BSCs observed in smoker patients. Secondly, cigarette smoking damages luminal airway cells [49,50], and BSCs could be activated to replenish differentiated airway cells. Consistently, studies in mice have shown that depletion of luminal airway cells results in the expansion of BSCs and their differentiation into secretory cells and ciliated cells [12]. We propose an additional mechanism by which the enhanced DNA repair capabilities of BSCs promote their proliferation after cigarette smoke exposure and could participate in smoking-induced basal cell hyperplasia.

Our findings provide evidence that BSCs are more proficient than alveolar cells in using NHEJ to repair their DNA. NHEJ has been implicated in the accumulation of genetic lesions [51] and plays a role in chromothripsis [52–54]—phenomena that participate in the initiation of tumour formation. Quiescent haematopoietic stem cells use NHEJ and displayed increased genomic instability after irradiation compared to progenitor cells, further implicating NHEJ in oncogenesis [38]. We propose the ability of BSCs to rapidly repair DNA through error-prone NHEJ allows the cells to survive longer and places them at greater risk than lung progenitor cells to accumulate mutations, which may ultimately lead to the induction of carcinogenesis (Fig 8).

In vivo studies in genetically modified mice have been used to demonstrate the cell of origin of cancer. AT2 cells were found to act as the tumour-initiating cells in K-Ras^G12D^-driven lung adenocarcinoma [35,36], whilst inactivation of *Tp53* and *Rb* specifically in lung neuroendocrine cells resulted in small cell lung cancer [55]. Based on its anatomical location in the upper airways and the expression of BSC markers, lung SqCC is thought to arise from BSCs. Surprisingly, a recent study in genetically modified mice showed that overexpression of *Sox2* in a *Cdkn2ab/Pten null* background could drive SqCC formation from BSCs, Club cells, or AT2 cells [56]. It remains to be seen whether multiple cells of origin are observed in other mouse models of lung SqCC, including mice with genetic backgrounds such as *Lkb1*^*-/-*^, *Lkb1*^*-/-*^*/Pten*^*-/-*^, or kinase-dead *Ikkα* [57–59]. In addition, such results may not be directly translatable in humans, given that mouse and human cells may not have the same degree of plasticity. Human tumours also carry much more genetic diversity than mouse cancer models, which is particularly relevant in lung cancer given its high levels of genomic instability [4]. To take into account the complexity of human cancers, computational comparison of normal cellular subset gene expression signatures to cancer subtypes has been used to gain insights into the cell of origin of human cancers [60,61]. Here we show that BSC gene expression signature closely resembles the human SqCC gene signature, suggesting that human lung BSCs are the candidate cells of origin of lung SqCC. An important caveat of such comparisons is the genetic signature of end-stage tumours may not fully represent the origin of the cancer. Our hypothesis will therefore need to be validated by introducing multiple genetic alterations in primary human lung cell subsets and determining their propensity for SqCC formation.

Invasive SqCC develops from preinvasive lesions in tobacco-smoking patients [62]. One-third of patients with basal cell hyperplasia will progress to carcinoma [63]; however, there are currently no biologic biomarkers to predict disease progression. Such biomarkers would greatly inform follow-up monitoring and perhaps enable early detection of invasive lesions. The discovery that BSCs are NHEJ competent and proliferate in response to DNA damage suggests that high levels of DNA-PKcs activation in cigarette smoking-induced basal cell hyperplasia may be a predictor of progression towards malignant disease. Assessment of the correlation between NHEJ activity in basal cell hyperplasia and progression to malignant disease would be necessary to validate this hypothesis. Advanced SqCCs are notoriously resistant to DNA-damaging agents [64]. Our data suggest that patients with strong expression of DNA repair genes such as *PRKDC* may benefit from therapy combining inhibitors of DNA repair and DNA-damaging agents. Overall, our study emphasizes the importance of fine-tuned control of DNA repair in stem/progenitor cells exposed to DNA-damaging agents, in which both unfaithful repair and failure to repair contribute to disease pathogenesis.

## Materials and Methods

### Human Samples and Mice

Adjacent normal lung specimens (confirmed by histology) were obtained through the Victorian Cancer Biobank from surgically resected tissue of lung cancer patients. Written informed consent was obtained from all patients by the Victorian Cancer BioBank prior to inclusion in the study, according to protocols approved by the Human Research Ethics Committee of the Walter and Eliza Hall Institute of Medical Research (WEHI) (approval #10/04). Patients were classified as current smokers (quit <10 y prior to surgery), exsmokers (quit >10 y), or never smokers (smoked less than 100 lifetime cigarettes). C57/Bl6 mice (8–12-wk-old males) were bred at the Walter and Eliza Hall Institute breeding facility, and SCID^*prkdc*^ mice (8–12-wk-old males) were obtained from the Animal Resource Centre (Australia). All animal experiments were approved by the WEHI Animal Ethics Committee (Approval #2013.028). Mice were maintained in our animal facilities according to institutional guidelines.

### Human Lung Cell Preparation

Lung tissue was classified as either large airway (LA, containing bronchi, cartilaginous airways, and attached alveolar tissue) or small airway (SA, containing bronchioles and attached alveolar tissue) and was processed either immediately or held intact for a maximum of 48 h at 4°C in DMEM/F12 media (Gibco) supplemented with 1 mg/mL of penicillin and streptomycin (Invitrogen). Samples were minced and then digested for 1 h at 37°C with 2 mg/mL collagenase (Worthington) and 200 U/mL deoxyribonuclease (Worthington) in 0.2% D-glucose (Sigma) in DPBS (Gibco). The cell suspension was strained through a 100 μm cell strainer and washed with 2% FCS-PBS, followed by red blood cell lysis to obtain a single-cell suspension.

### Human Lung Staining and FACS Sorting

Cells were blocked with rat immunoglobulin and CD16/CD32 FCγ II and III antibody (WEHI Monoclonal Antibody Facility) for 10 min at 4°C, followed by incubation with CD45-PE (HI30, BD Pharmingen), CD235a-PE (GA-R2, BD Pharmingen), CD140b-PE (28D4, BD Pharmingen), CD31-PE (WM59, BD Pharmingen), EpCAM-FITC (VU-1D9, Stem Cell Technologies), CD49f-PE-CY7 (GoH3, eBioscience), CD166-biotin (105902, R&D systems), and Podoplanin-APC (NC-08, BioLegend, also known as T1α) for 25 min at 4°C. The cells were then stained with streptavidin-APC-Cy7 (BD Pharmingen) before being washed and resuspended in 0.5 μg/mL propidium iodide. The cells were sorted on an Aria cytometer (BD Biosciences) using a 100 μm nozzle and processed immediately after sorting.

### Intracellular FACS Staining

Human cells were prepared and stained as for sorting using LIVE/DEAD Aqua (Life Technologies) as a viability marker. Cells were fixed and permeabilised using the BD Fix/Perm kit (BD Biosciences) and stained with keratin-5 (polyclonal, Covance), MUC5AC (45M1, Thermo Scientific), acetylated-tubulin (6-11B-1, Sigma Aldrich), SCGB1A1 (polyclonal, Millipore), or phospho-histone H2A.X Ser139-BV421 (γH2AX, N1-431, BD Horizon) antibodies. Cells were stained where appropriate with either anti-rabbit Alexa594 or anti-mouse Alexa594 (Molecular Probes) before analysis on a LSR Fortessa (BD Biosciences). For 4′,6-diamidino-2-phenylindole (DAPI) nuclear content analysis, cells were subjected to an additional fixation step in Permeabilise Plus buffer according to the manufacturer’s protocol (BD Biosciences) and deoxyribonuclease treatment (Worthington) before staining with DAPI. All analyses were performed using FlowJo software.

### Transmission Electron Microscopy

Sorted cells were immediately fixed in 2.5% glutaraldehyde and postfixed in osmium tetroxide according to standard electron microscopy protocols. The cells were subsequently embedded in EPON Araldite resin. Ultra-thin sections were cut on a Leica UCT ultramicrotome, stained with lead citrate and uranylacetate, and imaged using a Gatan Ultrascan camera on a Hitachi H-7500 transmission electron microscope.

### In Vitro Colony-Forming Assay

Freshly sorted cells were resuspended in DMEM/F12 (Gibco) supplemented with 1 mg/mL penicillin/streptomycin (Invitrogen), B27 (Gibco), 4 μg/mL heparin (Sigma Aldrich), 100 ng/mL EGF (Sigma Aldrich), insulin-transferrin-selenium (Gibco), 50 ng/mL human fibroblast growth factor-10 (R&D Systems), and 25 ng/mL human hepatocyte growth factor (R&D Systems), hereafter referred to as base media. A 50:50 matrigel (BD Biosciences):base media mix in a 96-well plate was allowed to set for 15 min at 37°C, before 2,000 cells were plated in media on top of the matrigel. Cells were grown for 14 d at 37°C in 5% CO_2_ and 5% O_2_ before colonies were photographed, counted, and processed for immunofluorescence studies.

### Immunofluorescence of Colonies in Matrigel

Matrigel colonies were fixed with 2% paraformaldehyde for 10 min at RT. Colonies were permeabilised with 0.3% TritonX in PBS for 10 min at 4°C then rinsed in 100 mM glycine in PBS. Blocking was performed with 10% goat serum in immunofluorescence buffer (0.1% BSA, 0.2% TritonX-100, and 0.05% Tween20 in PBS) followed by primary antibody staining with keratin-5 (polyclonal, Covance) or pro-SFTPC (polyclonal, Millipore) antibodies. Secondary antibodies were anti-rabbit Alexa594 or Alexa488 (Molecular Probes). Counterstaining of nuclei was performed using DAPI (Sigma Aldrich). The colonies were mounted on SuperFrost slides before 3-D imaging with a laser-scanning confocal microscope (Zeiss LSM 780). All imaging analyses were performed using Fiji software.

### γ-Irradiation

Whole fragments of human lungs were either nonirradiated (control) or γ-irradiated at 6 Gy and left to recover in DMEM/F12 media supplemented with 1 mg/mL of penicillin and streptomycin for 1 or 24 h post irradiation at 37°C in 5% CO_2_ and 5% O_2_. At each time point, tissue portions were harvested and fixed in 10% neutral buffered formalin (Sigma) overnight at room temperature before paraffin embedding and sectioning for immunostaining. For mouse immunofluorescence studies, mice (C57Bl/6, SCID^*prkdc*^) were exposed to 6 Gy of irradiation. Tracheas and lungs were harvested at 1, 4, 8, 24, or 96 h post irradiation and fixed/inflated in 4% paraformaldehyde in PBS pH 7.4 overnight at 4°C. Tracheas and lungs were then embedded in paraffin and sectioned for immunostaining. For mouse FACS analysis of cell cycle and apoptosis 24 h post IR, mice were irradiated and immediately injected with bromodeoxyuridine (BrdU, 50 mg/kg, Amersham). For FACS analysis of γH2AX expression, mouse lungs and tracheas were harvested 1 h or 4 h post irradiation, and single-cell suspensions generated as described below. This timing corresponds to 4 h or 7 h post irradiation including the time taken to generate single-cell suspensions.

### Bleomycin

Trachea and lungs from mice (C57/Bl6, SCID^*prkdc*^) were harvested at 1, 4, 8, 24, or 96 h after bleomycin (Hospira) administration (IV 40 mg/kg) before fixation, sectioning, and immunostaining as described above. For FACS analysis of γH2AX expression, mouse lungs and tracheas were harvested 1 h post injection, and single-cell suspensions generated as described below. This timing corresponds to 4 h post bleomycin injection including the time taken to generate single-cell suspensions.

### Mouse Lung Cell Preparation

Lungs were minced and then digested in 2 mg/mL collagenase in 0.2% glucose-PBS for 45 min at 37°C. Red blood cells were lysed (0.64% NH_4_Cl) and cells filtered through a 40 μm cell strainer to obtain a single-cell suspension. Mouse lung cells were blocked as described for human cells and stained with CD45-PE-Cy7 (30-F11, BioLegend), CD31-PE-Cy7 (390, BioLegend), EpCAM-APC-Cy7 (G8.8, BioLegend), and CD104-FITC (346-11A, BioLegend). Alveolar cells were identified as CD45^-^CD31^-^EpCAM^hi^CD104^lo^ as described previously [15]. Cells were then fixed and subjected either to Brdu/7-AAD staining (BD BrdU Flow Kit) according to the manufacturer’s instructions to identify apoptotic and proliferating cells or to γH2AX (20E3, Cell Signalling) staining.

### Mouse Tracheal Cell Preparation

Tracheal epithelial cells were isolated according to the protocol from Rock et al. [12]. Briefly, tracheas were cut into four pieces and incubated in 16 U/mL dispase (Roche) for 40 min at room temperature. Digestion was stopped with the addition of 5% FCS-DMEM (Gibco), and the epithelium peeled from the trachea. Epithelial sheets were washed and incubated in 2X trypsin-EDTA (Gibco) for 20 min at 37°C. Cells were then washed with 5% FCS-DMEM. Cells were blocked and stained with anti-NGFR (Abcam) or anti-T1α (clone 8.1.1, DHSB) antibodies for 30 min at 4°C. Cells were incubated with anti-rabbit Alexa Fluor488 or anti-hamster Alexa Fluor647 (Molecular Probes) for 15 min at 4°C. BSCs were identified as NGFR^+^ or T1α^+^ cells. Cells were then stained with BrdU, 7-AAD, or γH2AX (γH2AX, 20E3, Cell Signaling Technology).

### Immunostaining

Antigen retrieval was performed using citrate buffer (10 mM, pH 6) or high-pH antigen retrieval solution (Vector). Sections were blocked in 10% goat serum and incubated with antibodies overnight at 4°C followed by fluorophore-conjugated antibody for immunofluorescence or HRP-conjugated secondary antibodies (Vector) for immunohistochemistry. Antibodies used were phospho DNA-PKcs-S2056 (Abcam) and phospho-histone H2AX Ser139 (γH2AX, 20E3, Cell Signaling Technology) on human and mouse tissue; RAD51 (14B4, Genetex), T1α (clone 8.1.1, DHSB), and cleaved caspase 3 Asp175 (5A1E, Cell Signaling Technology) on mouse tissue; and T1α (NC-08, Biolegend) on human tissue. Nuclei were counterstained with DAPI where appropriate. Images were acquired using a DeltaVision fluorescence microscope (Applied Precision).

### RNA Sequencing and Quantitative PCR (qPCR)

Human and mouse RNA was extracted from snap-frozen sorted cell pellets using a RNeasy Micro Kit (Qiagen), and DNase treatment was performed using the TURBO DNA-free Kit (Ambion) according to the manufacturer’s instructions. RNA sequencing was performed on an Illumina HiSeq at the Australian Genome Research Facility. Per human sample, 16–26 million 100 bp single-end reads were generated, and 13–17 million 100 bp single-end reads were generated per mouse sample. For human qPCR analyses, cDNA was generated using the SuperScript III system (Life Technologies) and subject to qRT-PCR using the Sensimix SYBR Hi-Rox kit (Bioline) on the Rotorgene RG-6000 (Corbett Research) under standard conditions. Three technical replicates were performed for each sample. Taqman gene expression assays were used for *MUC5AC* (Hs0087365_mH) and *FOXJ1* (HS00230964_m1) using *18S* (HS99999901_s1) or *GAPDH* (HS99999905_m1) as reference genes (Life Technologies). The sequence of the primers is available in S1 Table.

### RNA-seq Analysis

Human RNA-seq reads were aligned to the hg19/GRCh37 genome, and mouse reads were aligned to the mm10 genome using Rsubread [65]. Reads were assigned to Entrez gene IDs using featureCounts [66] and Rsubread’s in-built RefSeq annotation. The raw sequence data and read counts are available from GEO series GSE83492 (human) and GSE83991 (mouse). Filtering and normalization used the edgeR package [67]. Genes were filtered if their counts per million (CPM) values were above 1 in fewer than three samples for the human data and above 0.2 in fewer than three samples for the mouse data. Library sizes were normalized by the trimmed mean of M-values (TMM) method [68]. Multidimensional scaling (MDS) plots were produced using edgeR’s plotMDS function with the default settings. Distances between points on the MDS plots represent leading fold change, the root-mean-square log2-fold change for the 500 genes that best distinguish each pair of RNA samples.

Differential expression analyses used the limma package [69]. Counts were transformed to log2-CPM values with associated precision weights using the voom function [70]. Gene set tests were performed using roast rotation gene set testing [71]. Signature genes were defined for each normal cell population to be those genes that were consistently either up- or down-regulated in that population versus every other cell population of the same species. Differential expression was assessed for this purpose using limma’s treat function with fold change thresholds varying from 1 to 1.2 and a false discovery rate of 0.05 [72]. Larger fold-change thresholds were used for populations with more signature genes. A log-fold change was associated with each signature gene, being the log2-fold change for that gene between the population for which it is a signature and the next closest population.

### CLCGP Microarray Data

Normalized microarray gene expression profiles for 261 lung cancers were downloaded from The Clinical Lung Cancer Genome Project (CLCGP) [33]. Gene symbols were converted to current official symbols with limma’s alias2SymbolTable function. Probes were filtered if their average log expression was in the bottom 50% or if no official symbol could be assigned. When more than one probe associated with the same gene, the probe with the highest average expression was retained. A signature score was computed for each normal lung cell population in each tumour profile: the signature scores were defined as Sum(w_g_ y_g_) / Sum(|w_g_|), where y_g_ is the log expression of the gene in the tumour and w_g_ is the log-fold change of the gene between the normal populations. The sums were taken over all signature genes for the normal population. Signature scores were scaled to be between 0 and 1 for each normal population. Barcode plots were created using limma’s barcodeplot function. Correlation between normal expression signatures and cancer subtypes was assessed using rotation gene set tests, with 9,999 rotations and with the normal cell log-fold changes as gene weights.

### TCGA RNA-seq Data

Genewise RNA-seq read counts for 125 lung adenocarcinomas, 224 SqCC, and 54 normal lung samples from The Cancer Genome Atlas (TCGA) project were obtained from GEO series GSE62944 [73]. Genes were filtered if they failed to achieve 0.1 CPM in at least 54 samples. TMM normalization was applied, and differential expression between the cancer subtypes and normal samples was assessed using limma-voom and moderated *t* tests [70]. The proportion of the genome altered for each of the lung SqCCs (derived from somatic copy number information) was downloaded from the TCGA data portal (http://cancergenome.nih.gov).

### Measurement of Telomere Length

Telomere lengths were determined as described previously [74]. Briefly, genomic DNA was extracted from snap-frozen FACS sorted human lung epithelial pellets or from 293T (ATCC) snap-frozen cell pellets using an Illustra Tissue and Cells GenomicPrep kit (GE Healthcare). The mastermix contained Sybr green I (0.75X, Life Technologies), AmpliTaq Gold (0.625 U, Life Technologies), Buffer II (1X, Life Technologies), MgCl_2_ (3 mM, Ambion), DTT (1 mM, Ambion), betaine (1 M, Sigma Aldrich), dNTPs (0.2 mM, Life Technologies), albumin primers (900 mM, IDT), and telomere primers (900 mM, IDT). The master mix was combined with 20 ng of experimental DNA and analysed in triplicate alongside a four-point 293T standard curve (50 to 1.85 ng of DNA) using albumin as the single-copy gene. PCR was performed on a Rotorgene RG-6000 using the following conditions: (15 min, 95°C) x 1, (15 s, 94°C − 15 s, 49°C) x 2, (15 s, 94°C − 10 s, 62°C −15 s, 74°C − 10 s, 84°C − 15 s, 88°C) x 32. Telomere lengths were calculated by determining the ratio of telomere signal (T) to single-copy gene signal (S) as determined from the 293T standard curve.

### Statistical Analysis

*p*-Values less than 0.05 were considered significant when conducting univariate tests. Error bars on plots represent mean ± SEM, and stars indicate significant differences in two-group comparisons: *p < 0.05, **p < 0.01, ***p < 0.001.

## Supporting Information

S1 Fig BSCs and AT2 cells form distinct clonal colonies. (A) The proportion of each epithelial cellular subset in human lung samples, as assessed by their percentage in lineage- (CD45, CD31, CD140b, CD235a) EpCAM+ cells. n = 20 patients for proximal lung samples and n = 24 patients for distal lung samples, 21–85 yo, male and female, never, ex- and current smokers. (B) Separated z-stack images of BSCs (upper) and AT2 cell colonies (lower) demonstrating their 3-dimensional structure. Colonies are immunostained for E-cadherin and DAPI. Representative image of colonies isolated from a 72 yo male, smoking status unknown. Scale bar, 100 μm. (C) Limiting dilution analysis of human lung progenitor colony forming capacity. n = 4 patients for BSC colonies (69–78yo, male and female, current and ex-smokers) and n = 9 for AT2 colonies (39–78yo, male and female, current, ex- and never-smokers). The underlying data for panel A and C can be found in the S1 Data file.
(TIF)

S2 Fig Colony forming capacity does not correlate with patient age, sex and time since quitting smoking. **A**) Linear regression analysis of the colony forming capacity of human lung BSCs and AT2 cells *vs* years since patient smoking cessation. A value of 100 indicates a patient has smoked less than 100 lifetime cigarettes, n = 19–22 patients, 21–83yo, male and female, current, ex- and never-smokers. (**B**) Linear regression analysis of BSCs or AT2 colony forming activity and patient age. n = 21–22 patients, 21–83yo, male and female, current, ex- and never-smokers. (**C**) Colony forming capacity of BSCs and AT2 cells by patient gender. n = 23–27 patients, 21–83yo, current, ex- and never-smokers. Data is mean ± SEM, Student’s *t* test. The underlying data for panels A, B and C can be found in the S1 Data file.
(TIF)

S3 Fig Human lung BSCs have longer telomeres than AT2 cells. (**A**) Heatmap of RNA-seq expression profiles of lung cancer cell populations from 3 patients (53–83yo males, current and ex-smokers). Z-scores are log2-RPKM values standardized by gene produced by edgeR, with a prior count of 3 to reduce variability at low counts, standardized to have mean 0 and standard deviation 1 for each gene. Plot shows 100 genes with the largest standard deviations. Samples are color-coded by cell population as for Fig 2C. Rows and columns are clustered by Euclidean distance and complete linkage. (**B**) Expression of *TERT* as detected by RNA sequencing in human lung epithelial populations, n = 3 patients (53–83yo males, current and ex-smokers). Expression values are reads per kilobase per million mapped reads (RPKM). Paired *t* test. (**C**) Telomere lengths of 6 patients (46–83yo, male and female, current and ex-smokers) relative to 293T cells, data represent the mean of three technical replicates per patient, paired *t* test. The underlying data for panel B and C can be found in the S1 Data file.
(TIF)

S4 Fig Mouse BSCs are poised to respond to DNA damage. (**A**) FACS gating strategy used for analysis and sorting of mouse lung alveolar cells and mouse tracheal BSCs. NGFR: nerve growth factor receptor. (**B**) Gene Ontology (GO) terms associated with DNA repair, proliferation and cell cycle are significantly up-regulated in BSCs compared to alveolar cells according to ROAST gene set tests (p<0.02). Each pair of bars corresponds to a GO term. Bars show the proportion of genes associated with the GO term that are more highly expressed in BSCs (orange) or alveolar cells (blue), as determined by limma’s roast function. (**C**) Immunofluorescence staining of γH2AX and T1α in alveoli or trachea of WT mice non-irradiated or 1hr post exposure to 0.5 Gy, 3 Gy or 6 Gy of ionizing radiation. T1α marks alveolar type 1 cells in the lung and BSCs in the trachea. Representative images for one of n = 3 mice at each dose. Scale bar 20μm. (**D**) Representative FACS plot showing the analysis of cell cycle and apoptosis (sub G1) in WT mouse lung alveolar and tracheal BSCs 24hr post irradiation (6 Gy). (**E**) Immunostaining for cleaved caspase 3 (CC3, apoptotic cells), T1α and DAPI (nuclei) in WT lung and trachea either non-irradiated, 4, 24 or 96hr post irradiation (6 Gy). Representative images for one of n = 3 mice at each time point. Scale bar 20μm.
(TIF)

S5 Fig Mouse BSCs use NHEJ to repair their DNA following bleomycin injury. (**A**) Expression of key non-homologous end joining (NHEJ) genes in mouse lung alveolar and mouse tracheal BSCs as detected by RNA sequencing, n = 3 WT mice for alveolar and BSCs. Paired *t* test. Expression values are reads per kilobase per million mapped reads (RPKM). (**B**) Representative FACS plots from WT and SCID^*prkdc*^ mice 0 and 4hr post bleomycin injection (40 mg/kg intravenously) showing γH2AX expression in lung alveolar cells (EpCAM^+^) and tracheal BSCs (T1α^+^). Timing corresponds to the number of hours between time of injection and generation of single cell suspension for FACS analysis. **(C)** Percentage of γH2AX-positive cells in WT and SCID^*prkdc*^ mice in EpCAM^+^ lung epithelial cells and T1α^+^ tracheal BSCs 0 and 4hr following bleomycin injection. n = 6 animals per group. Student’s *t* test. Timing corresponds to the number of hours between time of injection and generation of single cell suspension for FACS analysis. **(D)** Immunostaining or cleaved caspase 3 (CC3, apoptotic cells), T1α and DAPI (nuclei) in SCID^*prkdc*^ lung either non-irradiated, 4, 24 or 96hr post irradiation (6 Gy). Representative images for one of n = 3 mice at each time point. Scale bar 20μm.
(TIF)

S6 Fig Gene signature analysis comparing lung epithelial cell types to lung cancer subtypes. (A) Multi-dimensional scaling plot of lung cancer expression profiles from The Clinical Lung Cancer Project. Squamous cell carcinomas (SqCC), small cell lung cancers (SCLC) and adenocarcinomas (ADC) are colour-coded. (B) Boxplots showing the signature expression scores of human proximal luminal cells in each lung tumor subtype. Each boxplot shows the range of luminal scores in that cancer subtype. Box width indicates sample size. (C) Boxplots showing the signature expression scores of human lung proximal AT2 cells by lung tumor subtypes. (D) Boxplots showing the signature expression scores of human distal luminal cells by lung tumor subtype. (E) Boxplots showing the signature expression scores of human distal AT2 cells by lung tumor subtype.
(TIF)

S7 Fig Lung squamous cell carcinomas express higher level of DNA repair genes than lung adenocarcinomas. (**A**) Gene ontology (GO) terms associated with DNA repair or cell cycle are significantly up-regulated in SqCC compared to ADCs according to ROAST gene set tests (p<0.02). Each pair of bars corresponds to a GO term. Bars show the proportion of genes associated with that GO term that are more highly expressed in SqCC (orange) or ADC (blue), as determined by limma’s roast function. (**B**) Violin plots showing expression levels of selected genes in normal lung tissue, lung adenocarcinomas (ADC) and lung squamous cell carcinomas (SqCC) from TCGA. Expression values are in log2 counts per million (CPM). Significance is determined by the moderated t-tests. The underlying data for panel A can be found in the S1 Data file.
(TIF)

S1 Table Primer sequence. (XLSX)

S1 Data Data supporting the figures. Data supporting the figures.
(XLSX)

## Abbreviations

γH2AX: phosphorylated histone 2AX
AT1: alveolar type 1
AT2: alveolar type 2
BSC: basal stem cell
CC3: cleaved caspase 3
CFU: colony-forming unit
CPM: counts per million
DAPI: 4′,6-diamidino-2-phenylindole
DNA-PKcs: DNA-dependent protein kinase catalytic subunit
DSB: double-strand break
EpCAM: epithelial cell adhesion molecule
FACS: fluorescence-activated cell sorting
FOXJ1: forkhead box J1
HR: homologous recombination
IR: ionizing radiation
KRT5: keratin 5
MUC5AC: mucin 5AC
NHEJ: nonhomologous end joining
qPCR: quantitative PCR
ROAST: rotation gene set test(s)
ROS: reactive oxygen species
SCGB1A1: secretoglobin 1A1
SCID: severe combined immune deficiency
SFTPC: surfactant protein C
SqCC: squamous cell carcinoma
SSB: single-strand break
TCGA: The Cancer Genome Atlas
WT: wild type
