# Supplementary figures and images for: Lung Basal Stem Cells Rapidly Repair DNA Damage Using the Error-Prone Nonhomologous End-Joining Pathway
Source: PLoS Biol. 2017 Jan 26;15(1):e2000731. doi: 10.1371/journal.pbio.2000731 (PMC5268430; doi:10.1371/journal.pbio.2000731)

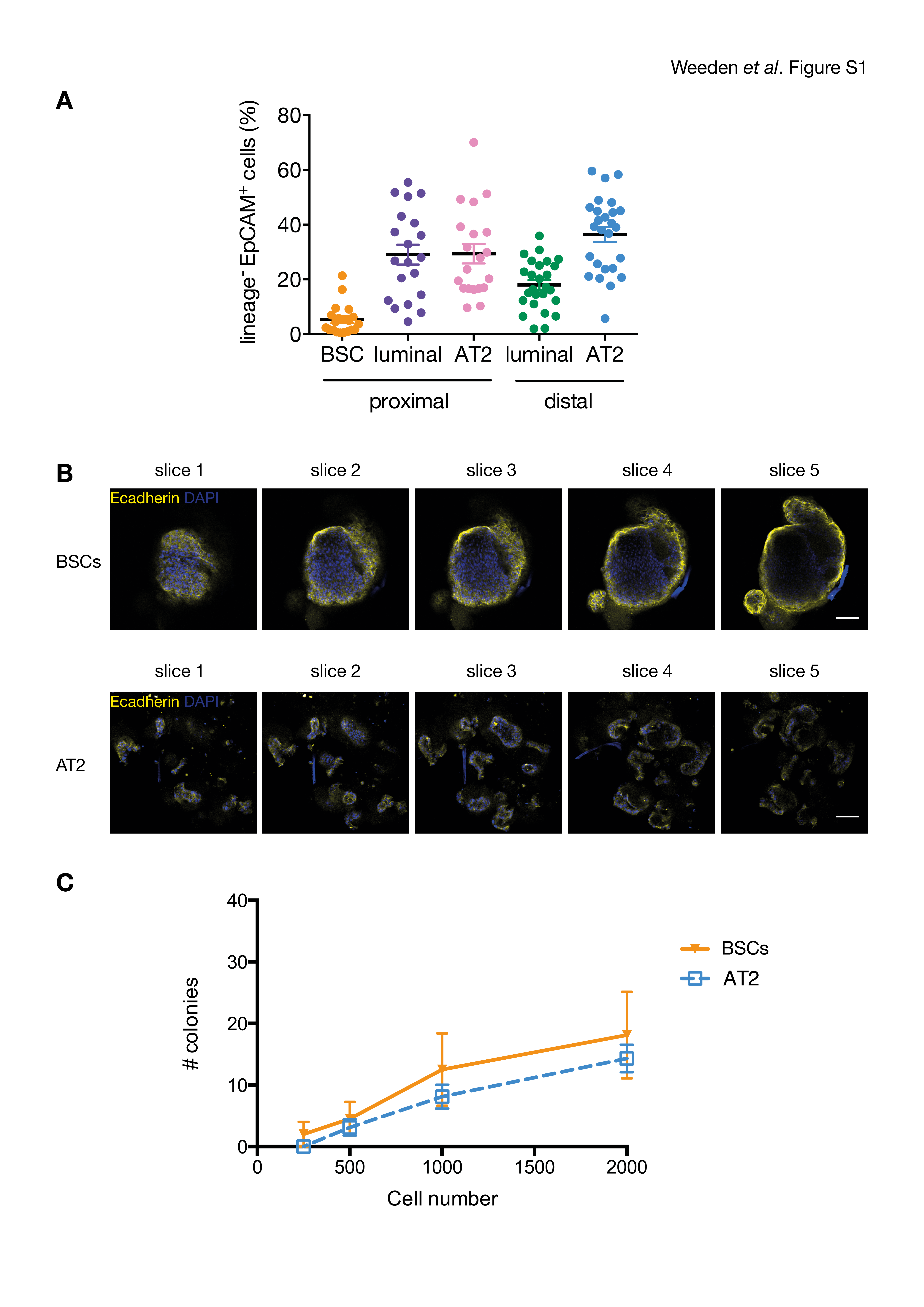

Supplement: S1 Fig — (A) The proportion of each epithelial cellular subset in human lung samples, as assessed by their percentage in lineage- (CD45, CD31, CD140b, CD235a) EpCAM+ cells. n = 20 patients for proximal lung samples and n = 24 patients for distal lung samples, 21–85 yo, male and female, never, ex- and current smokers. (B) Separated z-stack images of BSCs (upper) and AT2 cell colonies (lower) demonstrating their 3-dimensional structure. Colonies are immunostained for E-cadherin and DAPI. Representative image of colonies isolated from a 72 yo male, smoking status unknown. Scale bar, 100 μm. (C) Limiting dilution analysis of human lung progenitor colony forming capacity. n = 4 patients for BSC colonies (69–78yo, male and female, current and ex-smokers) and n = 9 for AT2 colonies (39–78yo, male and female, current, ex- and never-smokers). The underlying data for panel A and C can be found in the S1 Data file. (TIF) [file pbio.2000731.s001.tif]

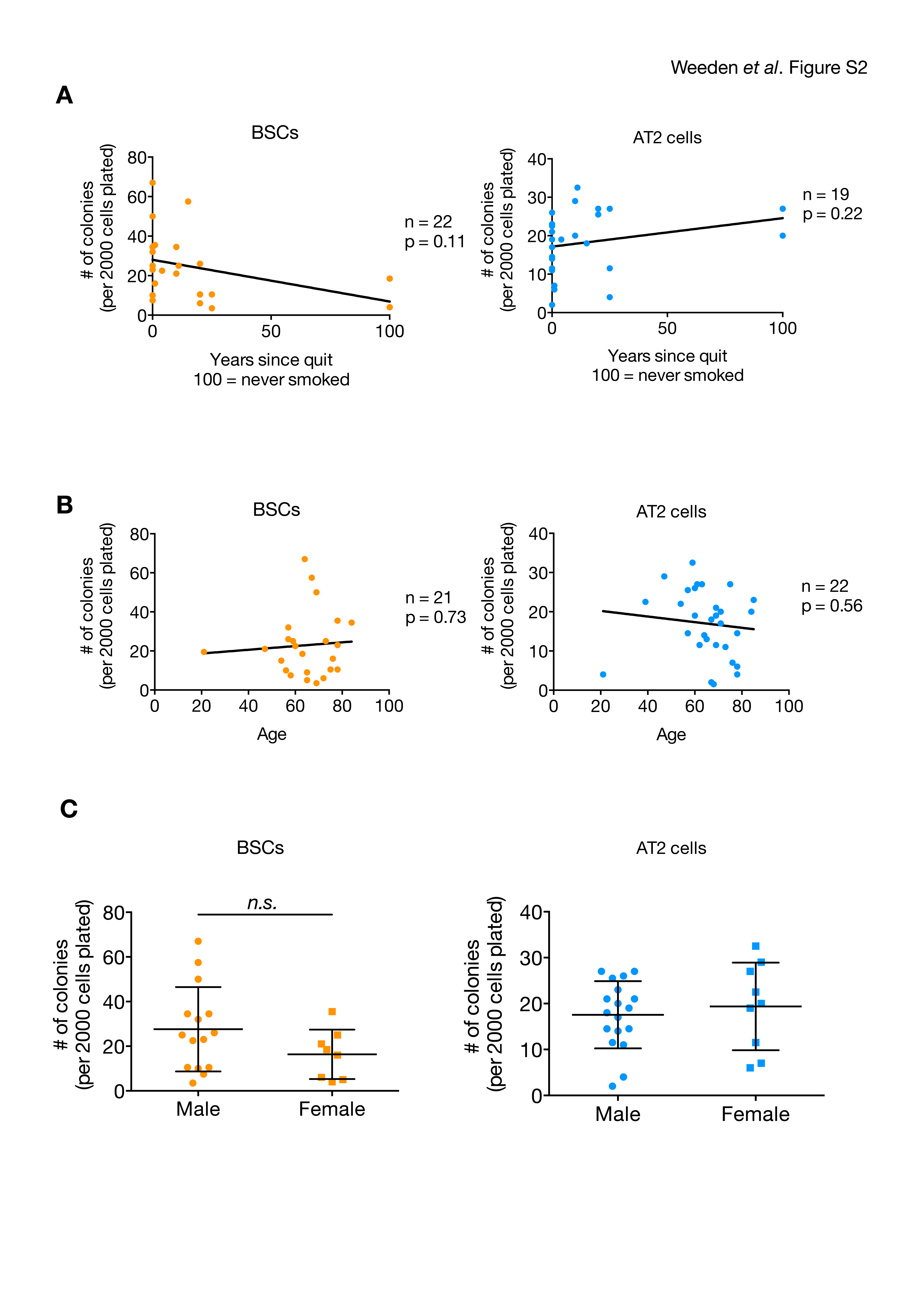

Supplement: S2 Fig — A) Linear regression analysis of the colony forming capacity of human lung BSCs and AT2 cells vs years since patient smoking cessation. A value of 100 indicates a patient has smoked less than 100 lifetime cigarettes, n = 19–22 patients, 21–83yo, male and female, current, ex- and never-smokers. (B) Linear regression analysis of BSCs or AT2 colony forming activity and patient age. n = 21–22 patients, 21–83yo, male and female, current, ex- and never-smokers. (C) Colony forming capacity of BSCs and AT2 cells by patient gender. n = 23–27 patients, 21–83yo, current, ex- and never-smokers. Data is mean ± SEM, Student’s t test. The underlying data for panels A, B and C can be found in the S1 Data file. (TIF) [file pbio.2000731.s002.tif]

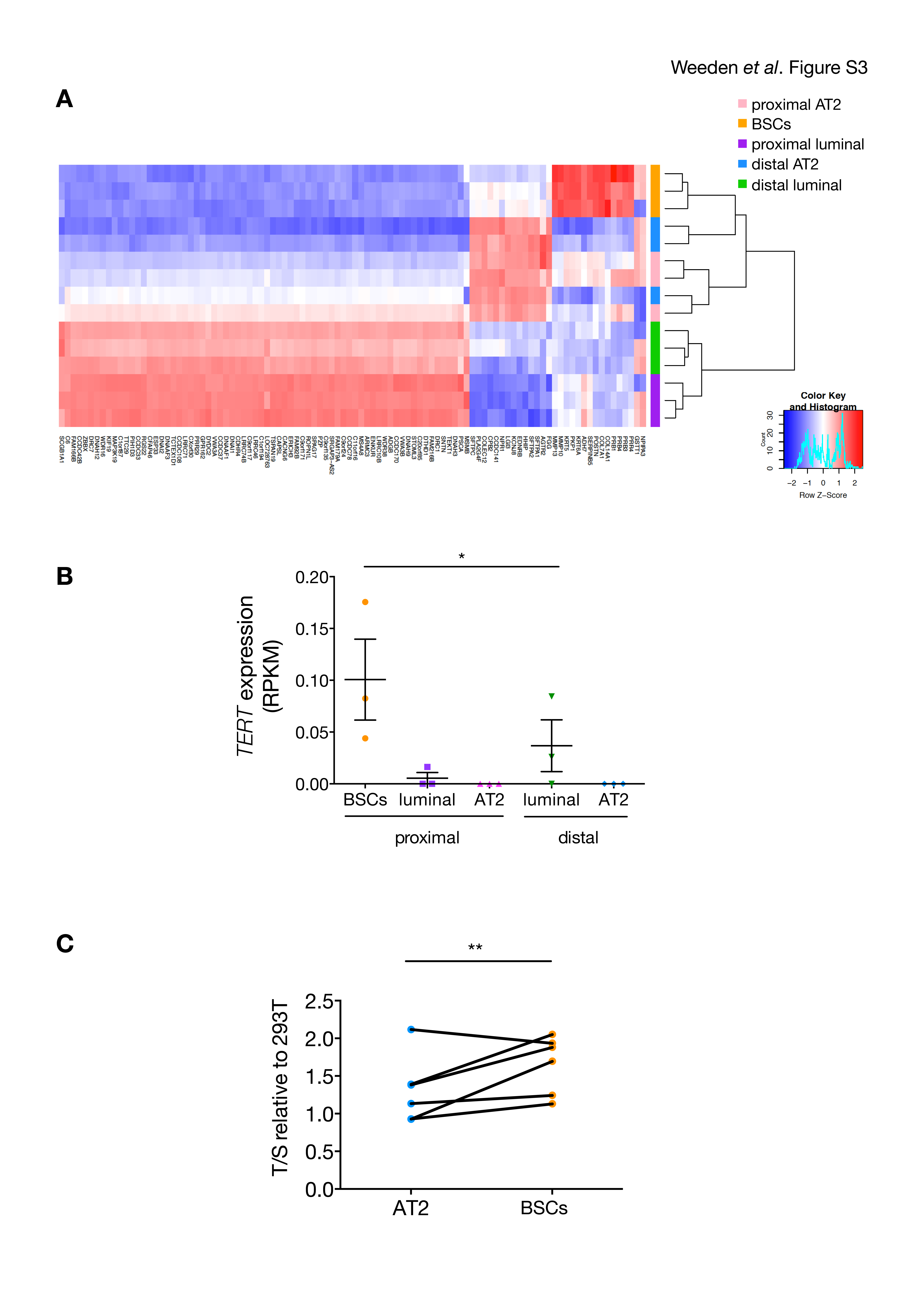

Supplement: S3 Fig — (A) Heatmap of RNA-seq expression profiles of lung cancer cell populations from 3 patients (53–83yo males, current and ex-smokers). Z-scores are log2-RPKM values standardized by gene produced by edgeR, with a prior count of 3 to reduce variability at low counts, standardized to have mean 0 and standard deviation 1 for each gene. Plot shows 100 genes with the largest standard deviations. Samples are color-coded by cell population as for Fig 2C. Rows and columns are clustered by Euclidean distance and complete linkage. (B) Expression of TERT as detected by RNA sequencing in human lung epithelial populations, n = 3 patients (53–83yo males, current and ex-smokers). Expression values are reads per kilobase per million mapped reads (RPKM). Paired t test. (C) Telomere lengths of 6 patients (46–83yo, male and female, current and ex-smokers) relative to 293T cells, data represent the mean of three technical replicates per patient, paired t test. The underlying data for panel B and C can be found in the S1 Data file. (TIF) [file pbio.2000731.s003.tif]

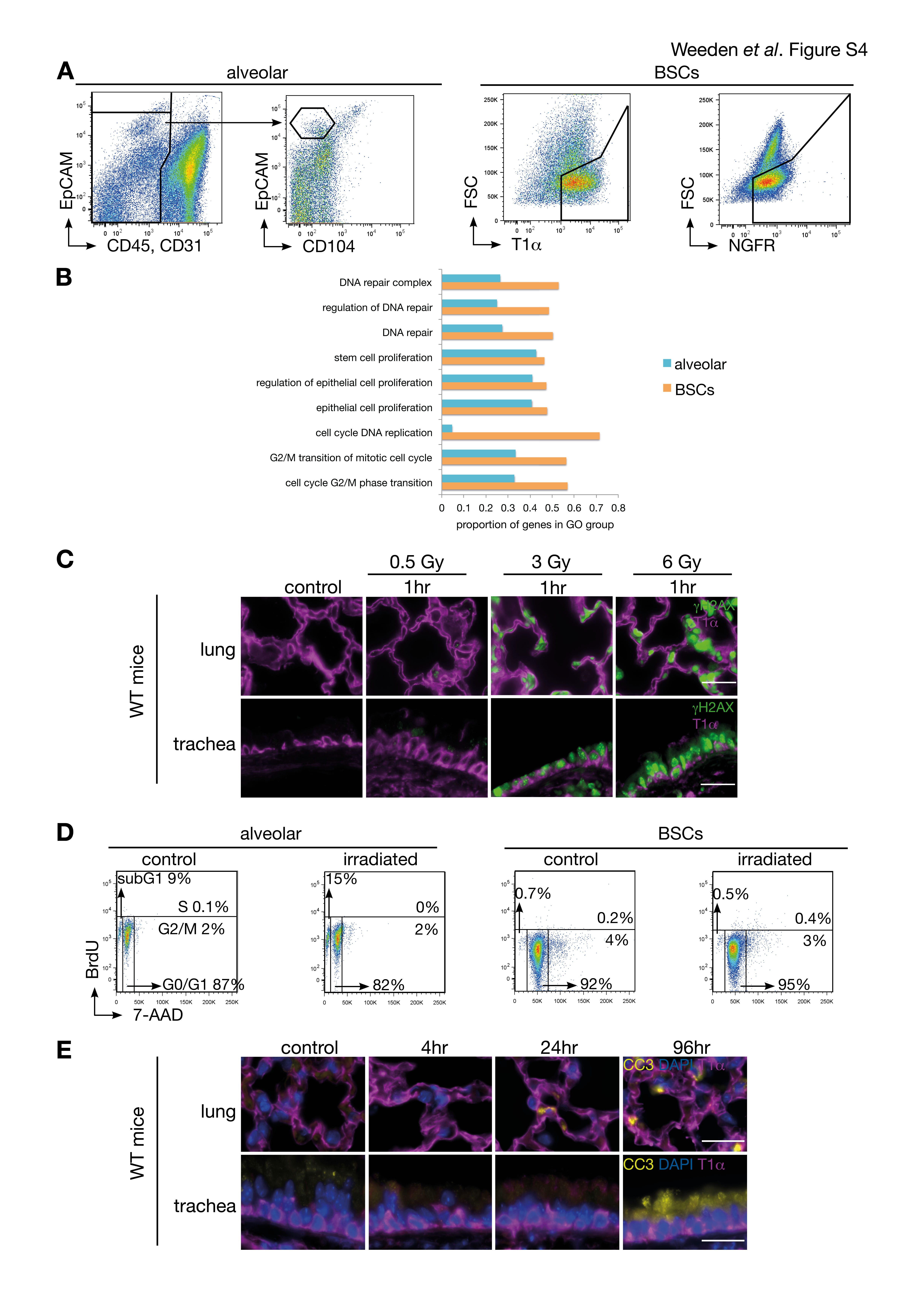

Supplement: S4 Fig — (A) FACS gating strategy used for analysis and sorting of mouse lung alveolar cells and mouse tracheal BSCs. NGFR: nerve growth factor receptor. (B) Gene Ontology (GO) terms associated with DNA repair, proliferation and cell cycle are significantly up-regulated in BSCs compared to alveolar cells according to ROAST gene set tests (p<0.02). Each pair of bars corresponds to a GO term. Bars show the proportion of genes associated with the GO term that are more highly expressed in BSCs (orange) or alveolar cells (blue), as determined by limma’s roast function. (C) Immunofluorescence staining of γH2AX and T1α in alveoli or trachea of WT mice non-irradiated or 1hr post exposure to 0.5 Gy, 3 Gy or 6 Gy of ionizing radiation. T1α marks alveolar type 1 cells in the lung and BSCs in the trachea. Representative images for one of n = 3 mice at each dose. Scale bar 20μm. (D) Representative FACS plot showing the analysis of cell cycle and apoptosis (sub G1) in WT mouse lung alveolar and tracheal BSCs 24hr post irradiation (6 Gy). (E) Immunostaining for cleaved caspase 3 (CC3, apoptotic cells), T1α and DAPI (nuclei) in WT lung and trachea either non-irradiated, 4, 24 or 96hr post irradiation (6 Gy). Representative images for one of n = 3 mice at each time point. Scale bar 20μm. (TIF) [file pbio.2000731.s004.tif]

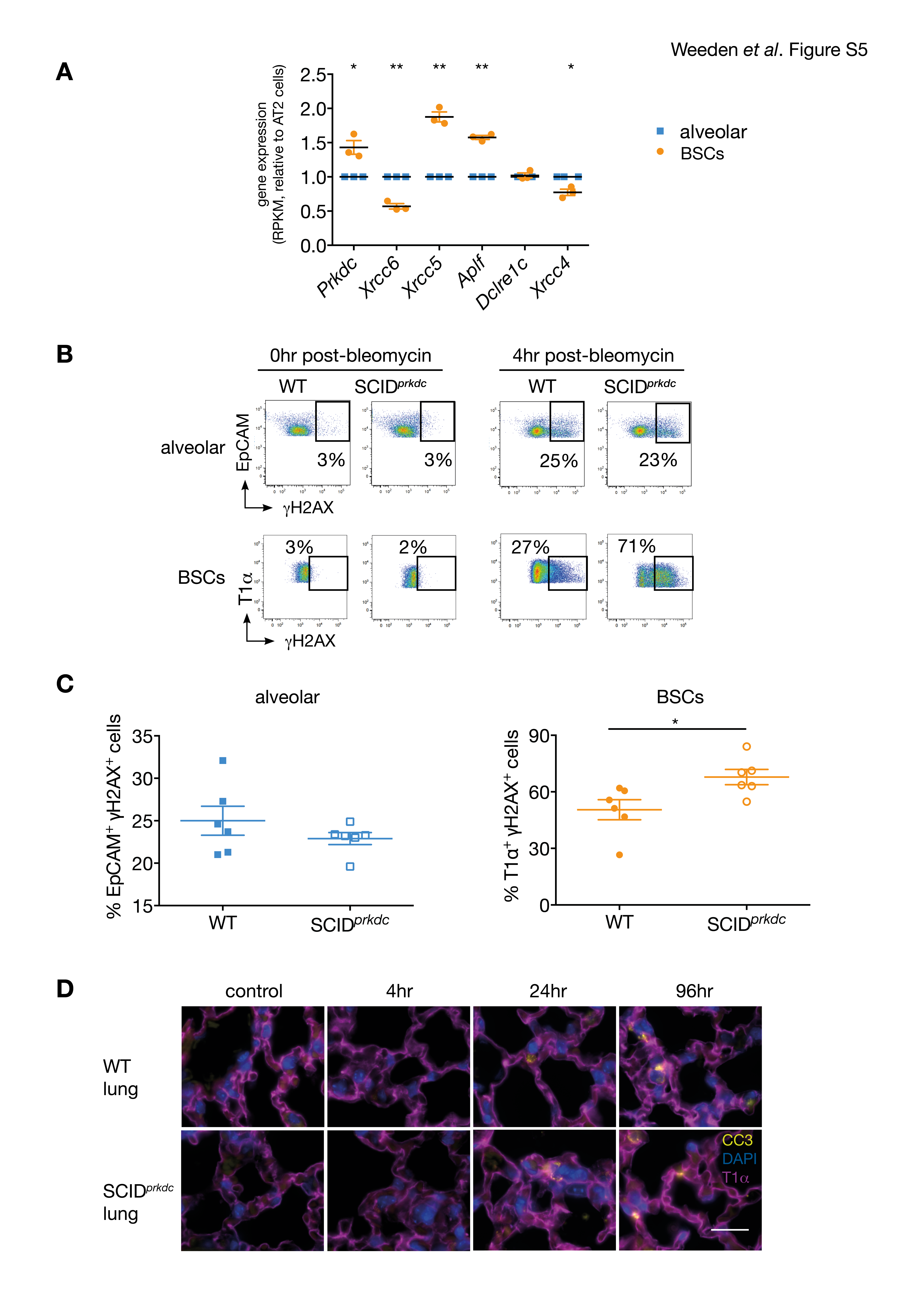

Supplement: S5 Fig — (A) Expression of key non-homologous end joining (NHEJ) genes in mouse lung alveolar and mouse tracheal BSCs as detected by RNA sequencing, n = 3 WT mice for alveolar and BSCs. Paired t test. Expression values are reads per kilobase per million mapped reads (RPKM). (B) Representative FACS plots from WT and SCIDprkdc mice 0 and 4hr post bleomycin injection (40 mg/kg intravenously) showing γH2AX expression in lung alveolar cells (EpCAM+) and tracheal BSCs (T1α+). Timing corresponds to the number of hours between time of injection and generation of single cell suspension for FACS analysis. (C) Percentage of γH2AX-positive cells in WT and SCIDprkdc mice in EpCAM+ lung epithelial cells and T1α+ tracheal BSCs 0 and 4hr following bleomycin injection. n = 6 animals per group. Student’s t test. Timing corresponds to the number of hours between time of injection and generation of single cell suspension for FACS analysis. (D) Immunostaining or cleaved caspase 3 (CC3, apoptotic cells), T1α and DAPI (nuclei) in SCIDprkdc lung either non-irradiated, 4, 24 or 96hr post irradiation (6 Gy). Representative images for one of n = 3 mice at each time point. Scale bar 20μm. (TIF) [file pbio.2000731.s005.tif]

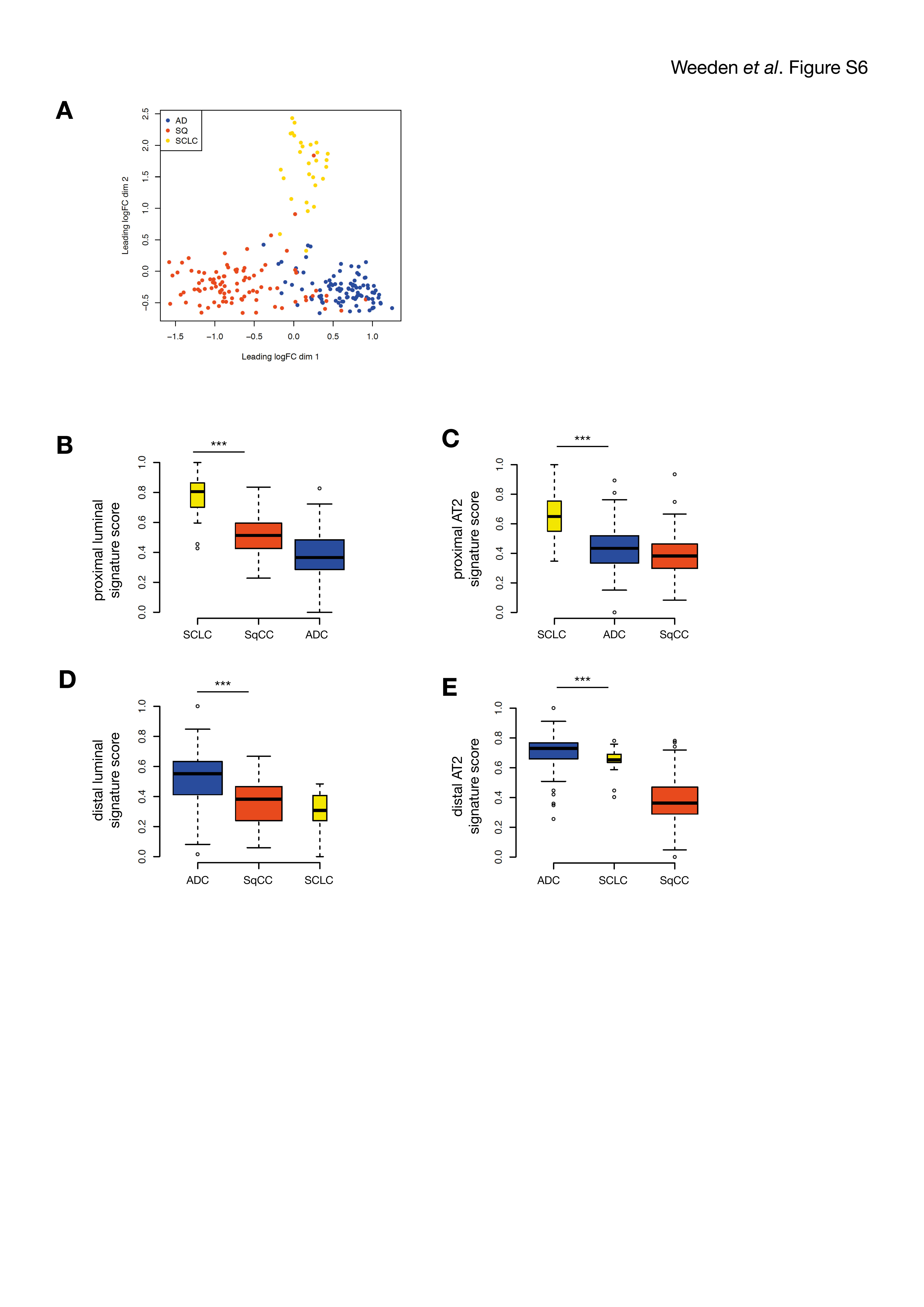

Supplement: S6 Fig — (A) Multi-dimensional scaling plot of lung cancer expression profiles from The Clinical Lung Cancer Project. Squamous cell carcinomas (SqCC), small cell lung cancers (SCLC) and adenocarcinomas (ADC) are colour-coded. (B) Boxplots showing the signature expression scores of human proximal luminal cells in each lung tumor subtype. Each boxplot shows the range of luminal scores in that cancer subtype. Box width indicates sample size. (C) Boxplots showing the signature expression scores of human lung proximal AT2 cells by lung tumor subtypes. (D) Boxplots showing the signature expression scores of human distal luminal cells by lung tumor subtype. (E) Boxplots showing the signature expression scores of human distal AT2 cells by lung tumor subtype. (TIF) [file pbio.2000731.s006.tif]

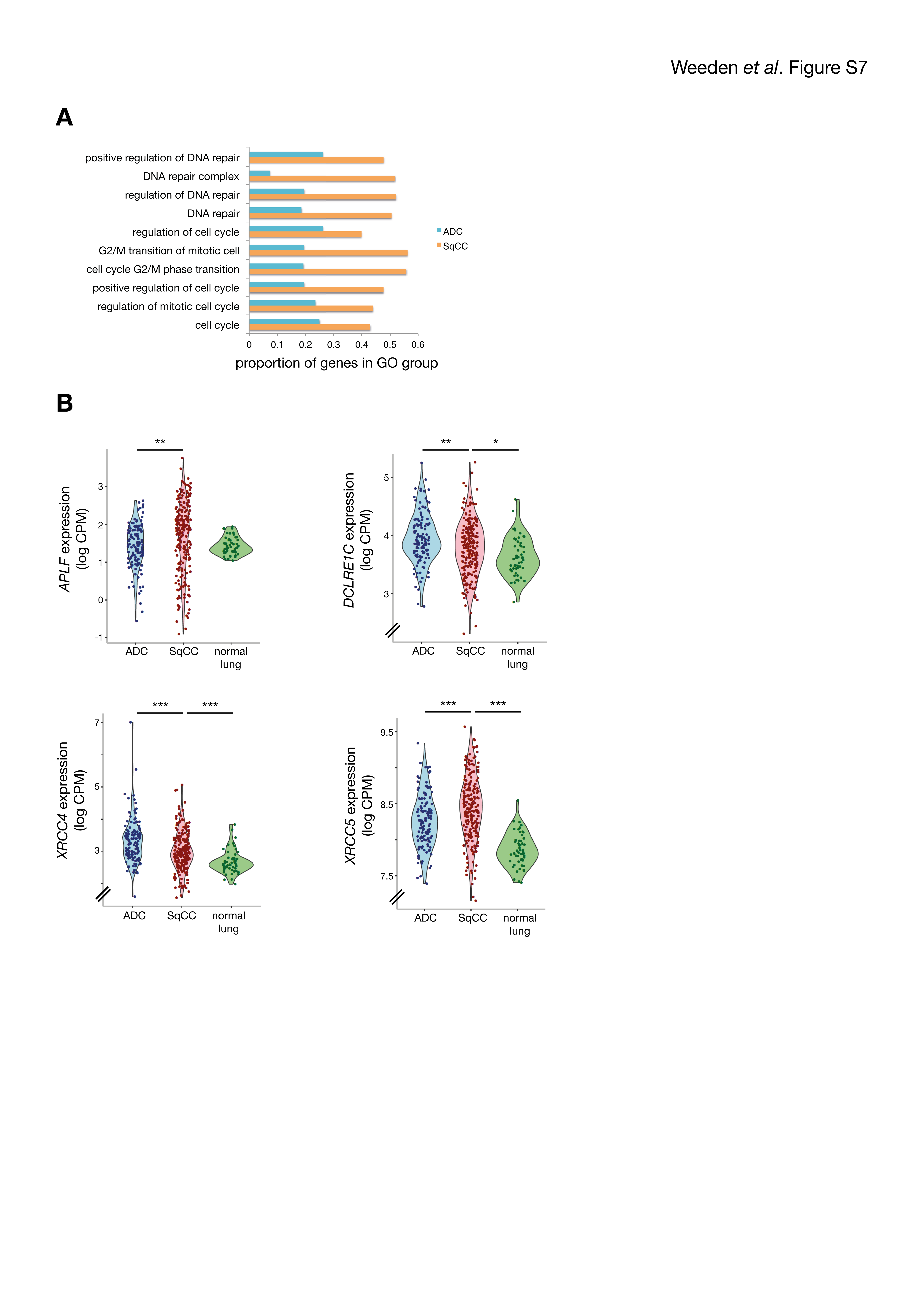

Supplement: S7 Fig — (A) Gene ontology (GO) terms associated with DNA repair or cell cycle are significantly up-regulated in SqCC compared to ADCs according to ROAST gene set tests (p<0.02). Each pair of bars corresponds to a GO term. Bars show the proportion of genes associated with that GO term that are more highly expressed in SqCC (orange) or ADC (blue), as determined by limma’s roast function. (B) Violin plots showing expression levels of selected genes in normal lung tissue, lung adenocarcinomas (ADC) and lung squamous cell carcinomas (SqCC) from TCGA. Expression values are in log2 counts per million (CPM). Significance is determined by the moderated t-tests. The underlying data for panel A can be found in the S1 Data file. (TIF) [file pbio.2000731.s007.tif]
